# Supplementary material for: Understanding solvent effects on adsorption and protonation in porous catalysts
Source: Nat Commun. 2020 Feb 26;11:1060. doi: 10.1038/s41467-020-14860-6 (PMC7044222; doi:10.1038/s41467-020-14860-6)
Supplement: Supplementary file 1 — Supplementary Information [file 41467_2020_14860_MOESM1_ESM.pdf]

Understanding Solvent Effects on Adsorption and Protonation in Porous Catalysts

Gould et al.

Supplementary Information for

Understanding Solvent Effects on Adsorption and Protonation in Porous Catalysts

Nicholas S. Gould[a], Sha Li[a], Hong Je Cho[a], Harrison Landfield[a], Stavros Caratzoulas[a], Dionisios Vlachos[a], Peng Bai[b,]* and Bingjun Xu[a,]*

[a]Catalysis Center for Energy Innovation, Department of Chemical and Biomolecular Engineering, University of Delaware, 150 Academy Street, Newark, DE 19716

[b]Department of Chemical Engineering, University of Massachusetts, Amherst, 686 North Pleasant Street, Amherst, MA 01003

*Email: pengbai@umass.edu, bxu@udel.edu

Supplementary Table 1. Langmuir fitted *K*' values for pyridine adsorption isotherms in Supplementary Figure 3, free energy change values at infinite dilution based on Supplementary Equation 1, and comparison to vacuum pyridine TPD.

Sample	MAS *K*'	BAS *K*'	MAS ΔG_{ads} Inf. Dil. (kJ mol^{-1})	Pyridinium Loss in Vacuum at 300 °C [18]
Si/ZSM-5	234	--	−15.1	--
H/ZSM-5 (36)	242	627	--	11 %
H/ZSM-5 (12)	207	521	--	22 %
Si/Beta (hydrophobic)	368	--	−16.6	--
Si/Beta (hydrophilic)	207	--	−15.0	--
H/Beta (12)	215	452	--	31 %

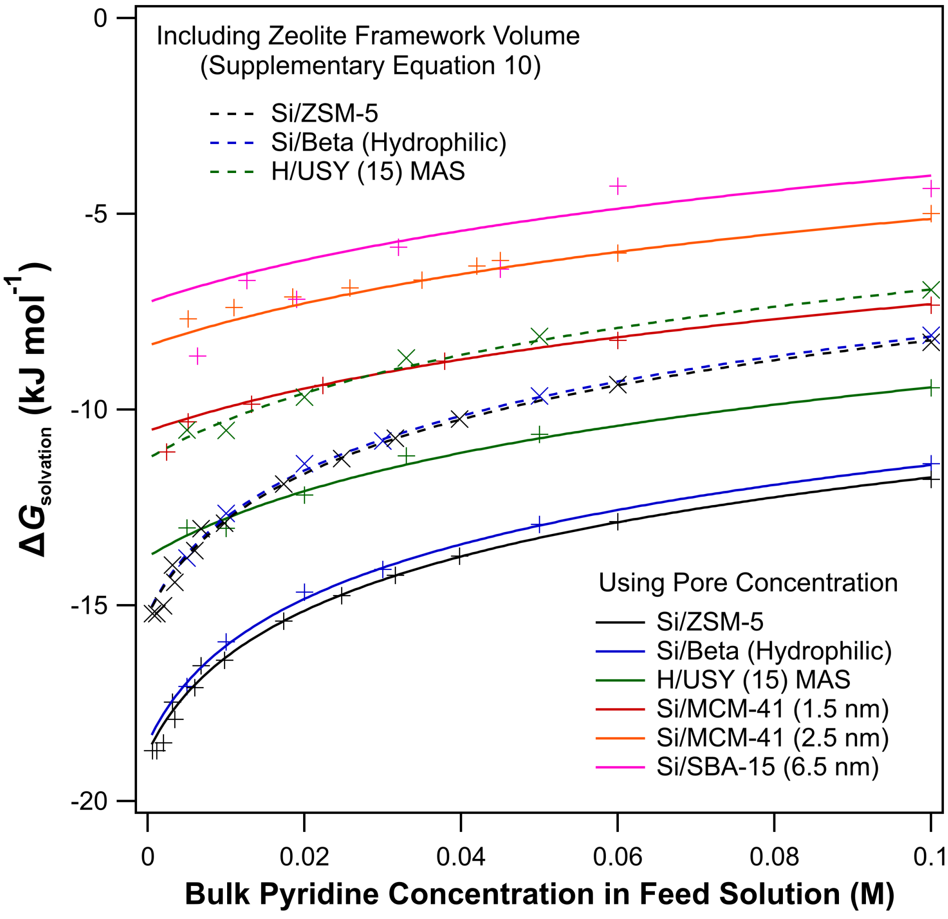

Supplementary Figure 1. Pyridine ΔG_{ads} on porous materials of varying average pore diameter in liquid water at 20 °C based on the isotherms in Figure 2A and Equation 1. Dashed lines use a value for the zeolite pore-phase concentration that includes the volume occupied by the solid, zeolite framework atoms (Equation 17). Solid lines are based upon the volume of micro/mesopores alone. Source data are provided as a Source Data file.

Supplementary Table 2. Henry's Law standard-state chemical potential differences at 20 °C and 1 bar.

Zeolite & Solvent	$\mu^{\circ}_{\mathrm{pyr,Z}} - \mu^{\circ}_{\mathrm{pyr,L}}$ (kJ mol^{-1})
ZSM-5 & Water	−15.25
USY & Water	−12.98
Beta & Water	−15.21
$\mu^{\circ}_{pyr,L} - \mu^{\circ}_{pyr,V}$ (Water)	−13.73

$\mu^{\circ}_{\mathrm{pyr,Z}}$ is 1 M pore phase pyridine based on extrapolation of dilute solution behavior.

$\mu^{\circ}_{\mathrm{pyr,L}}$ is 1 M liquid pyridine based on extrapolation of dilute solution behavior.

$\mu^{\circ}_{\mathrm{pyr,V}}$ is pure ideal gas pyridine at 1 bar.

Using volume correction including framework volume.

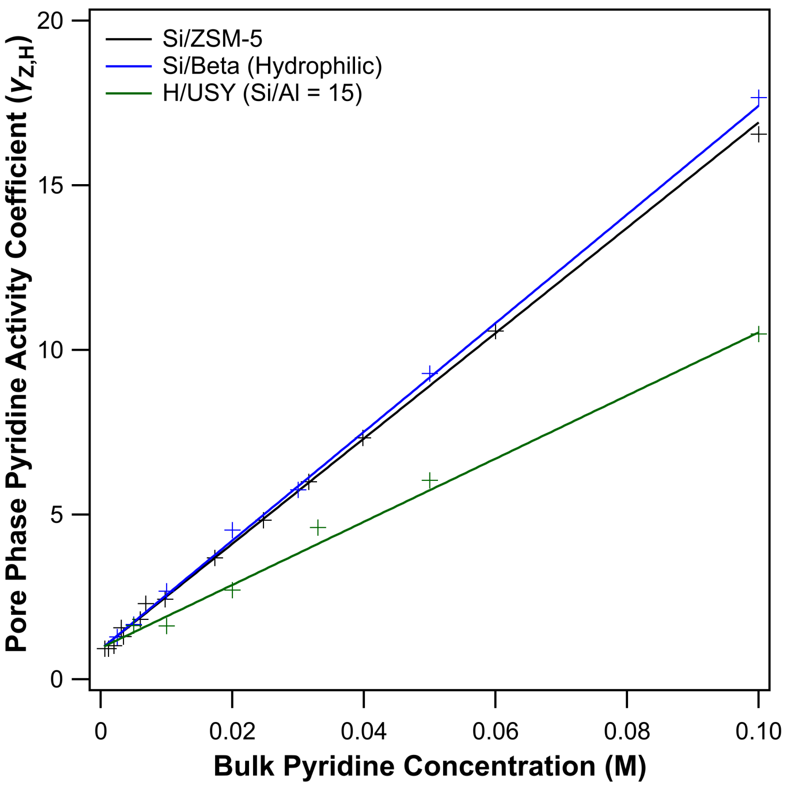

Supplementary Figure 2. Plot of activity coefficient vs. pyridine concentration in various zeolites. Pore-phase pyridine activity coefficients on porous materials of varying average pore diameter in liquid water at 20 °C based on the isotherms in Figure 2A. Source data are provided as a Source Data file.

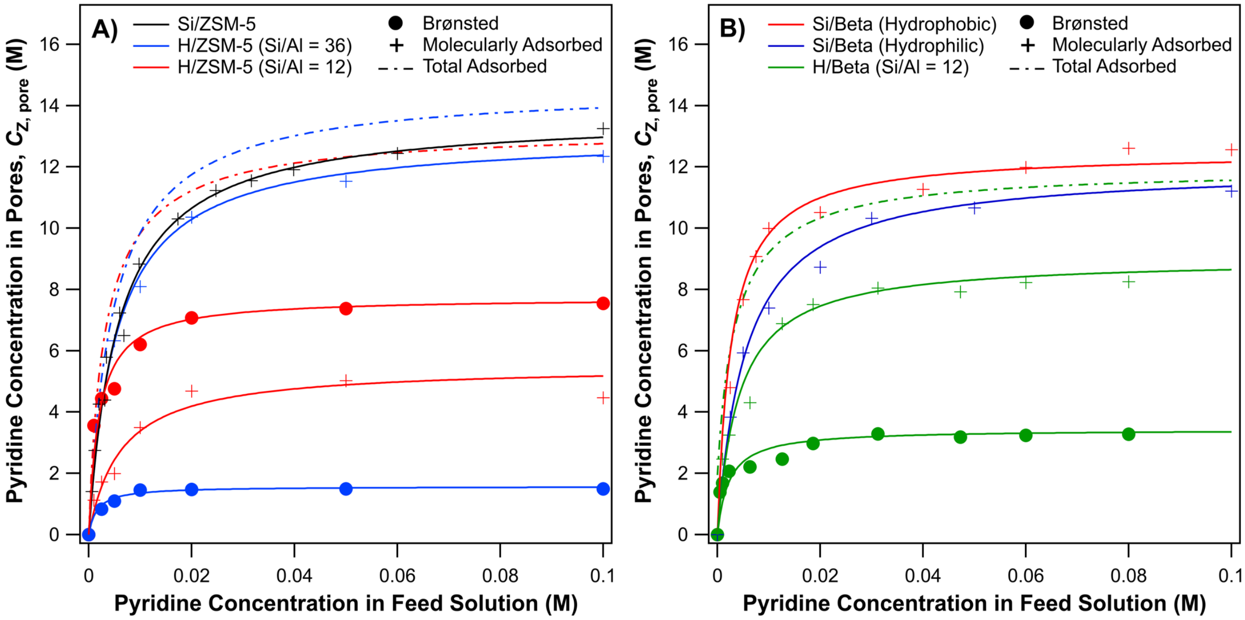

Supplementary Figure 3. Pyridine adsorption isotherms on zeolites. Pyridine adsorption isotherms on ZSM-5 (A) and Beta (B) samples in liquid water in ATR-FTIR at 20 °C. Source data are provided as a Source Data file.

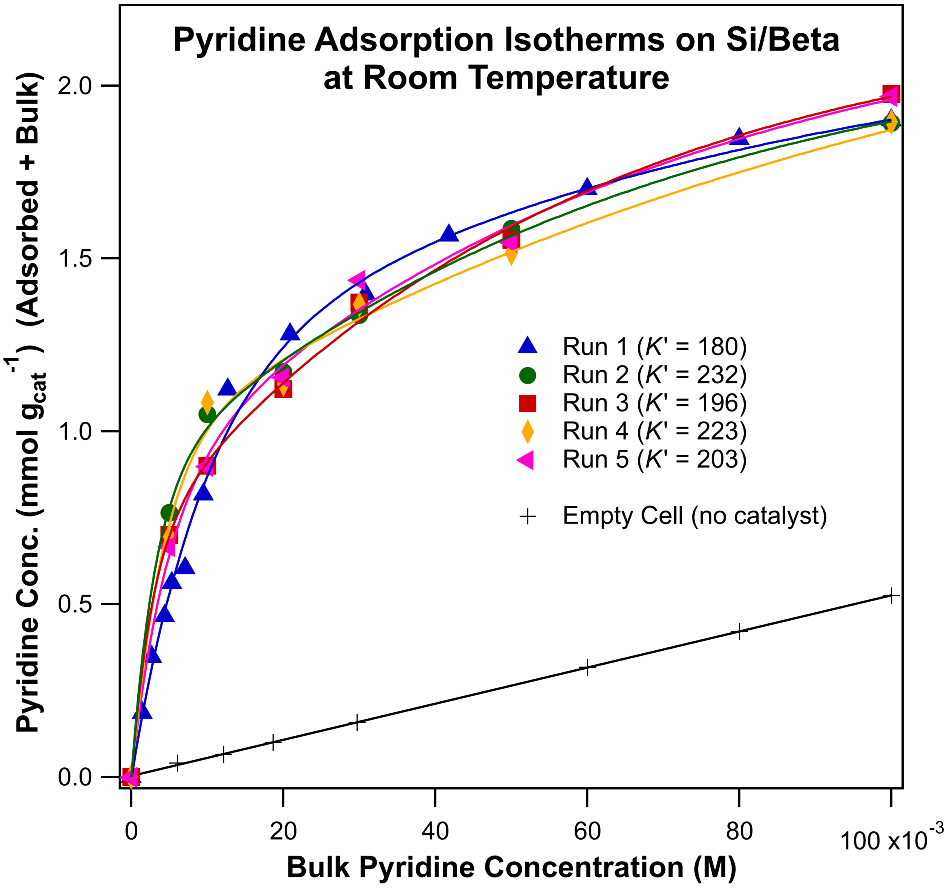

Supplementary Figure 4. Reproducibility of pyridine adsorption isotherms. Liquid phase adsorption isotherms of pyridine into Si/Beta (hydrophilic) at 20 °C repeated 5 times. The control experiment of the empty cell area in black (bulk pyridine contribution). Source data are provided as a Source Data file.

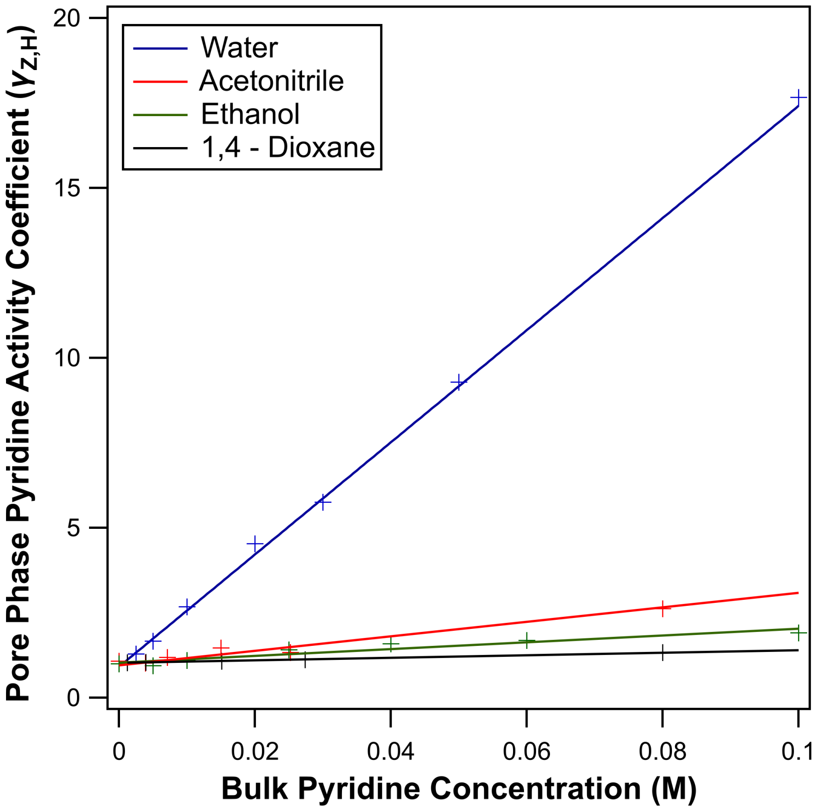

Supplementary Figure 5. Pyridine activity coefficient in different solvents. Pore-phase pyridine activity coefficients in Si/Beta at 20 °C in various solvents based on the isotherms in Figure 3. Source data are provided as a Source Data file.

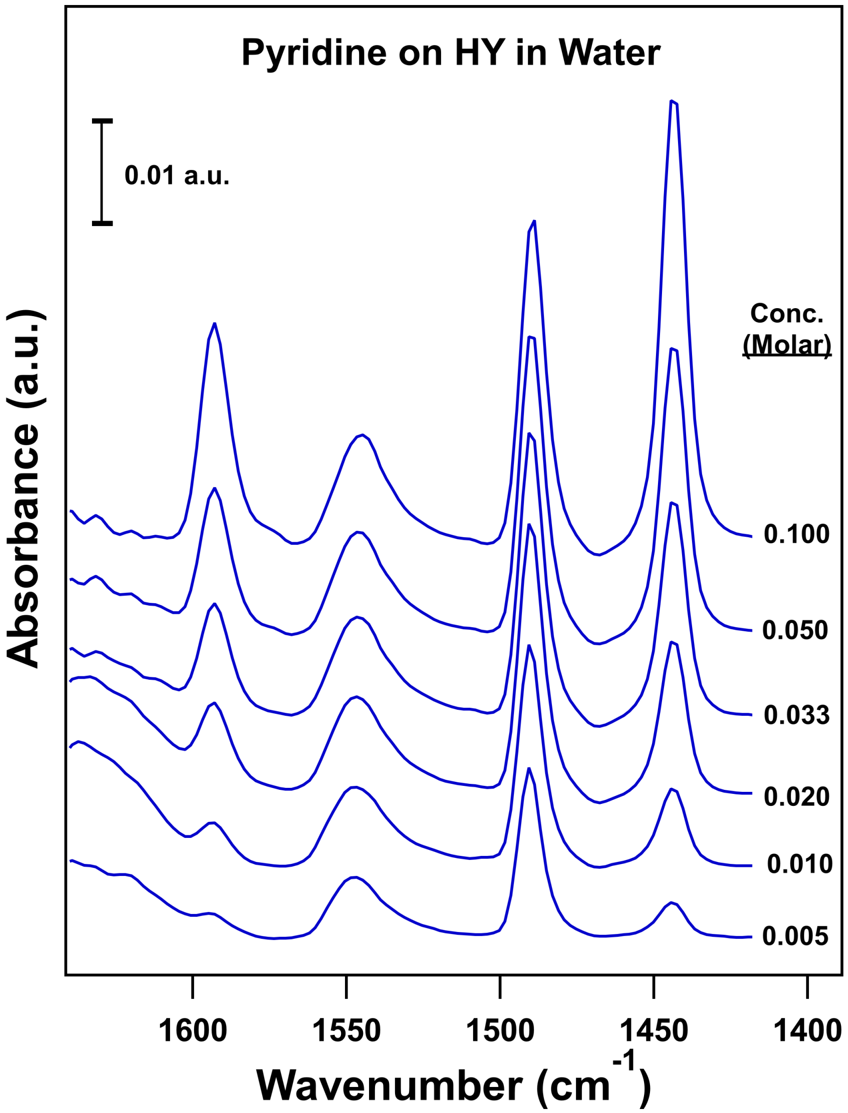

Supplementary Figure 6. ATR-FTIR spectra of pyridine adsorbed on zeolites. ATR-FTIR spectra of pyridine adsorbed on H/Y (Si/Al = 3) in liquid water at 20 °C.

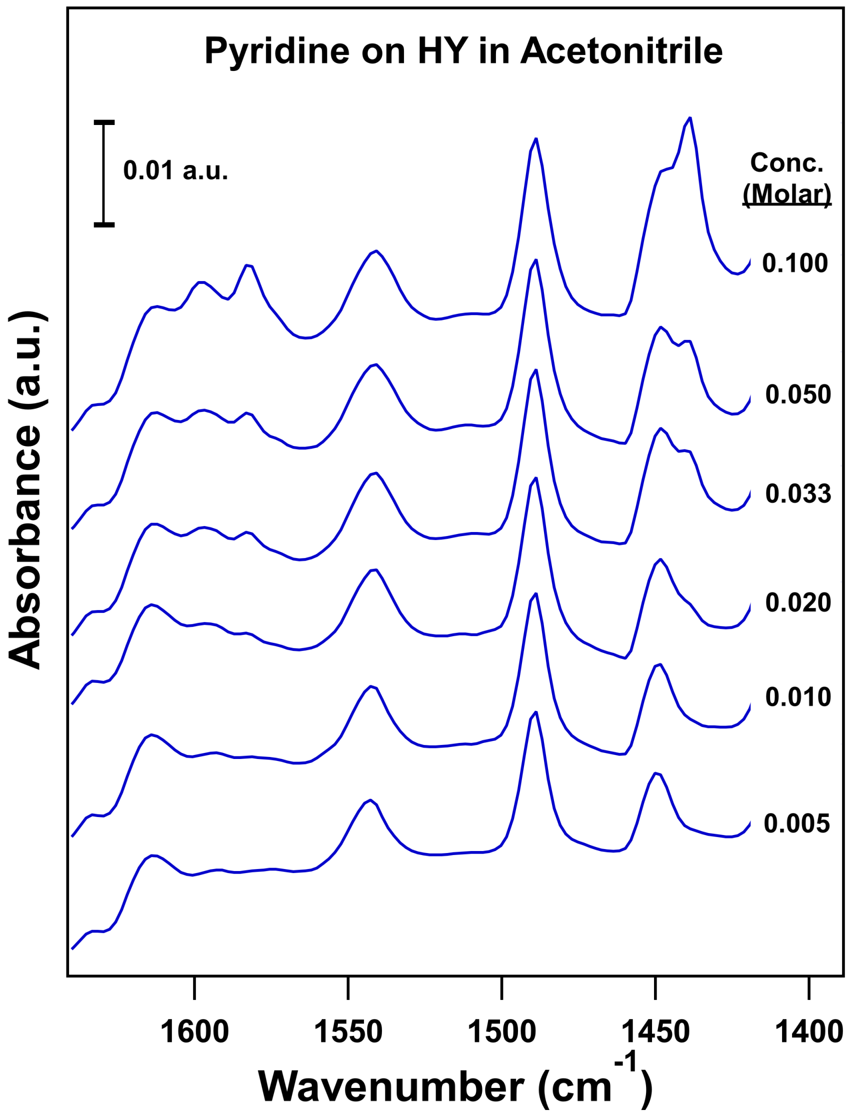

Supplementary Figure 7. ATR-FTIR spectra of pyridine adsorbed on zeolites. ATR-FTIR spectra of pyridine adsorbed on H/Y (Si/Al = 3) in liquid acetonitrile at 20 °C.

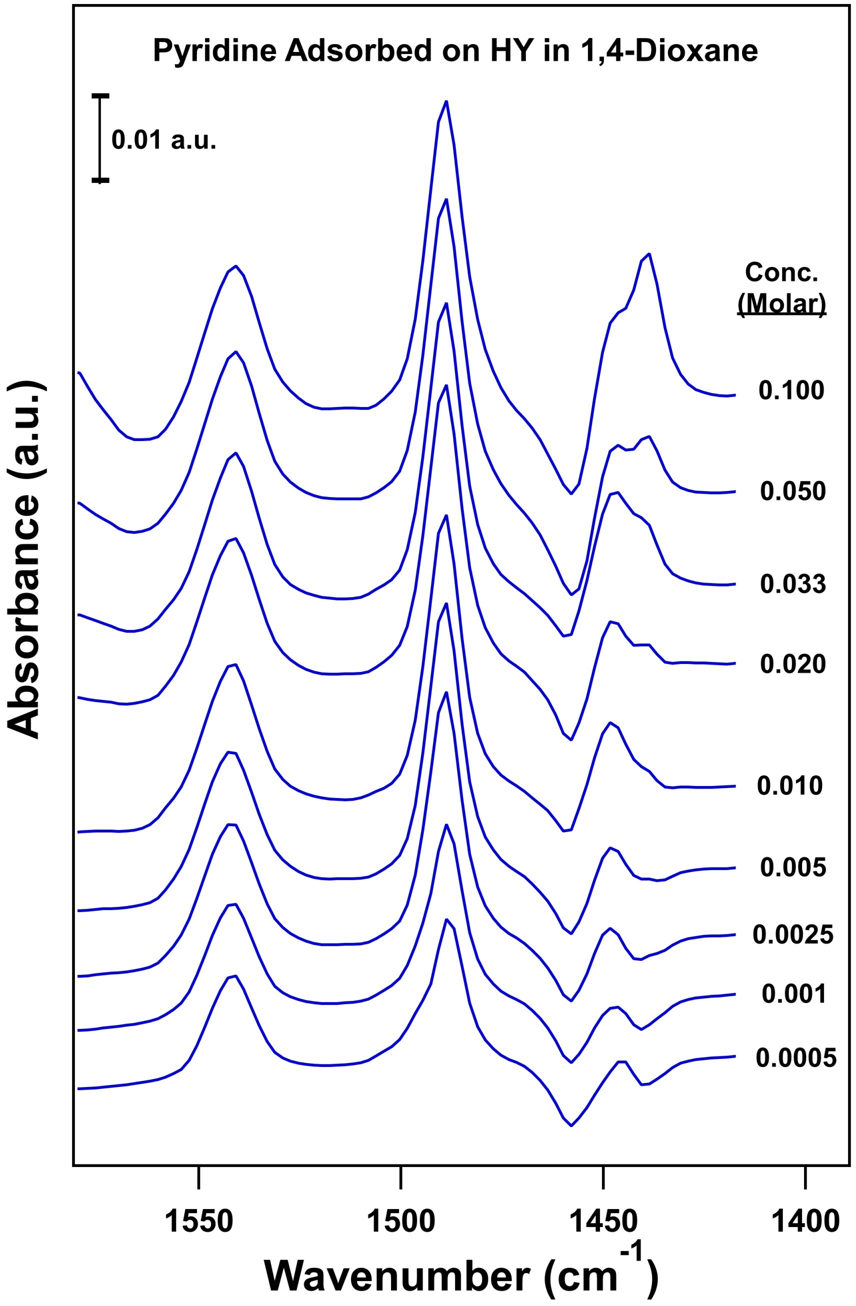

Supplementary Figure 8. ATR-FTIR spectra of pyridine adsorbed on zeolites. ATR-FTIR spectra of pyridine adsorbed on H/Y (Si/Al = 3) in liquid 1,4 - dioxane at 20 °C.

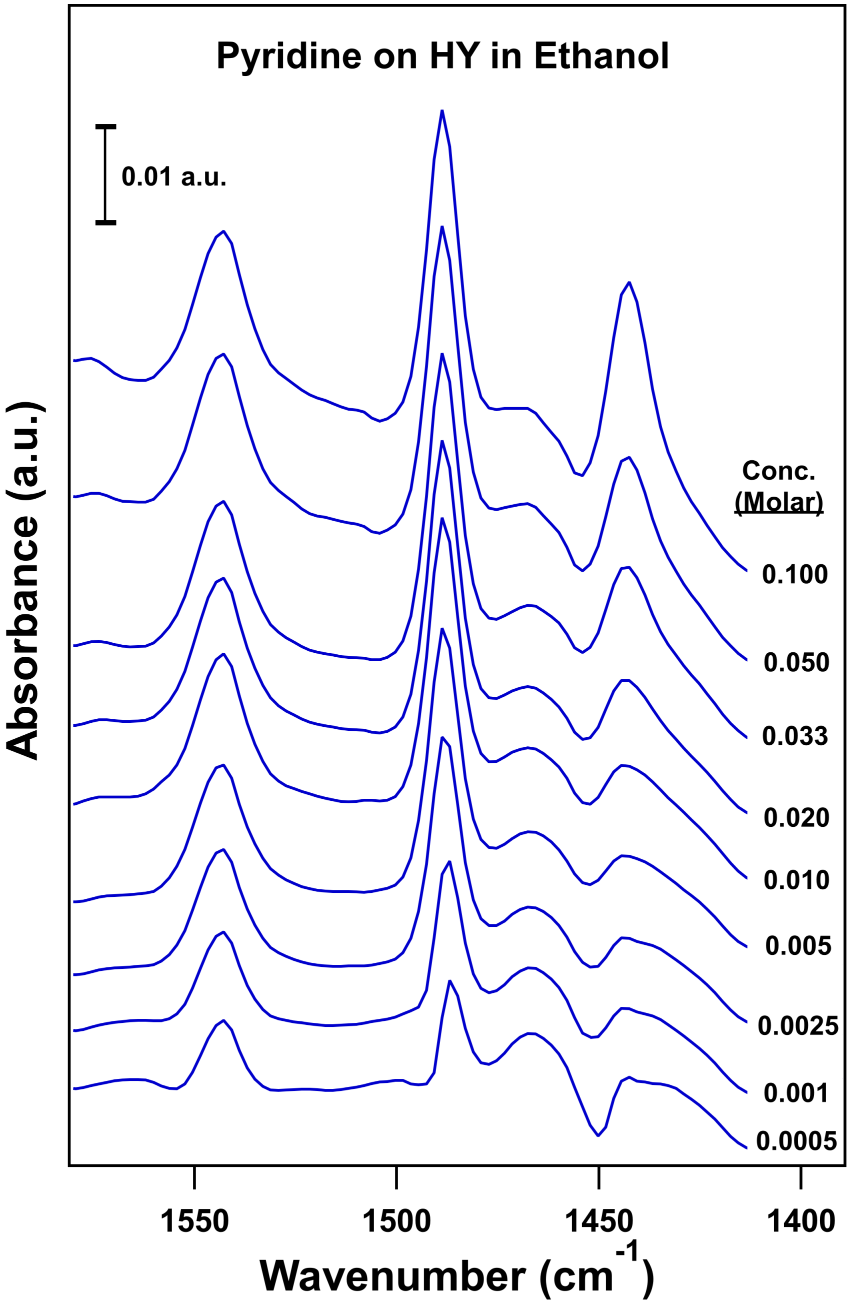

Supplementary Figure 9. ATR-FTIR spectra of pyridine adsorbed on zeolites. ATR-FTIR spectra of pyridine adsorbed on H/Y (Si/Al = 3) in liquid ethanol at 20 °C.

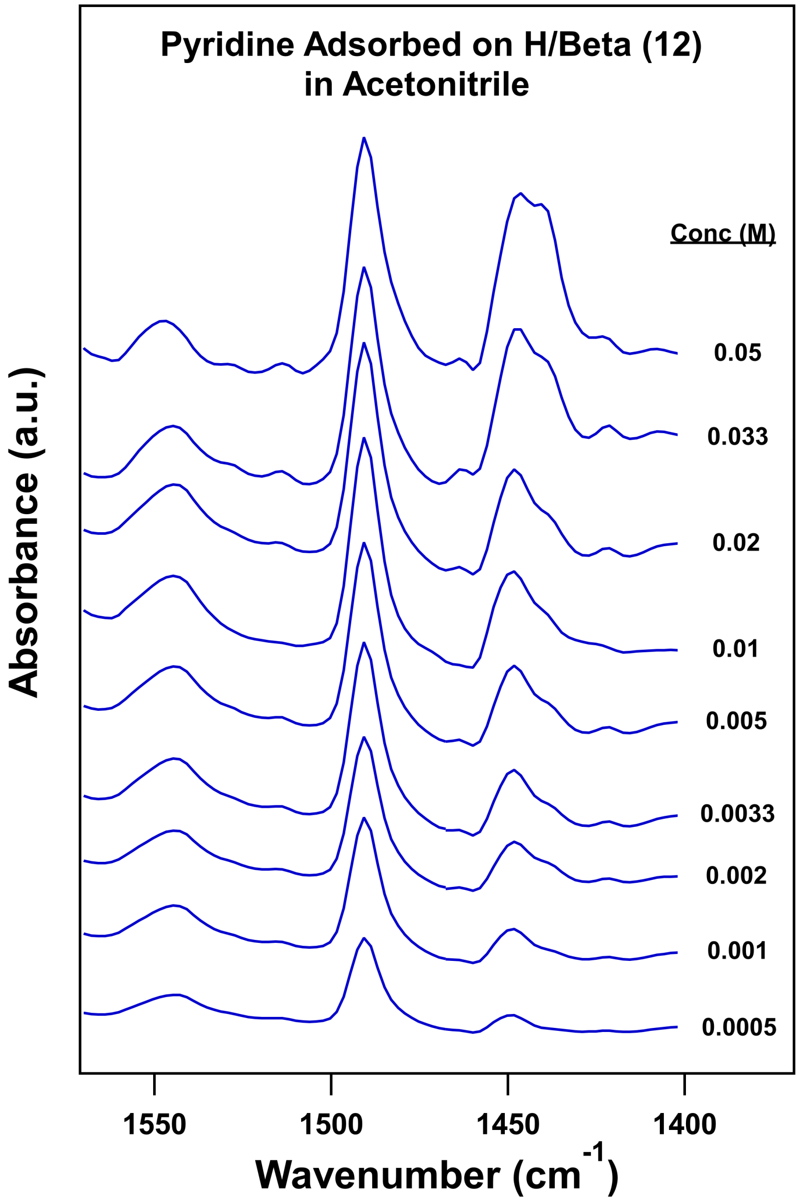

Supplementary Figure 10. ATR-FTIR spectra of pyridine adsorbed on zeolites. ATR-FTIR spectra of pyridine adsorbed on H/Beta (Si/Al = 12) in liquid acetonitrile at 20 °C. Source data are provided as a Source Data file.

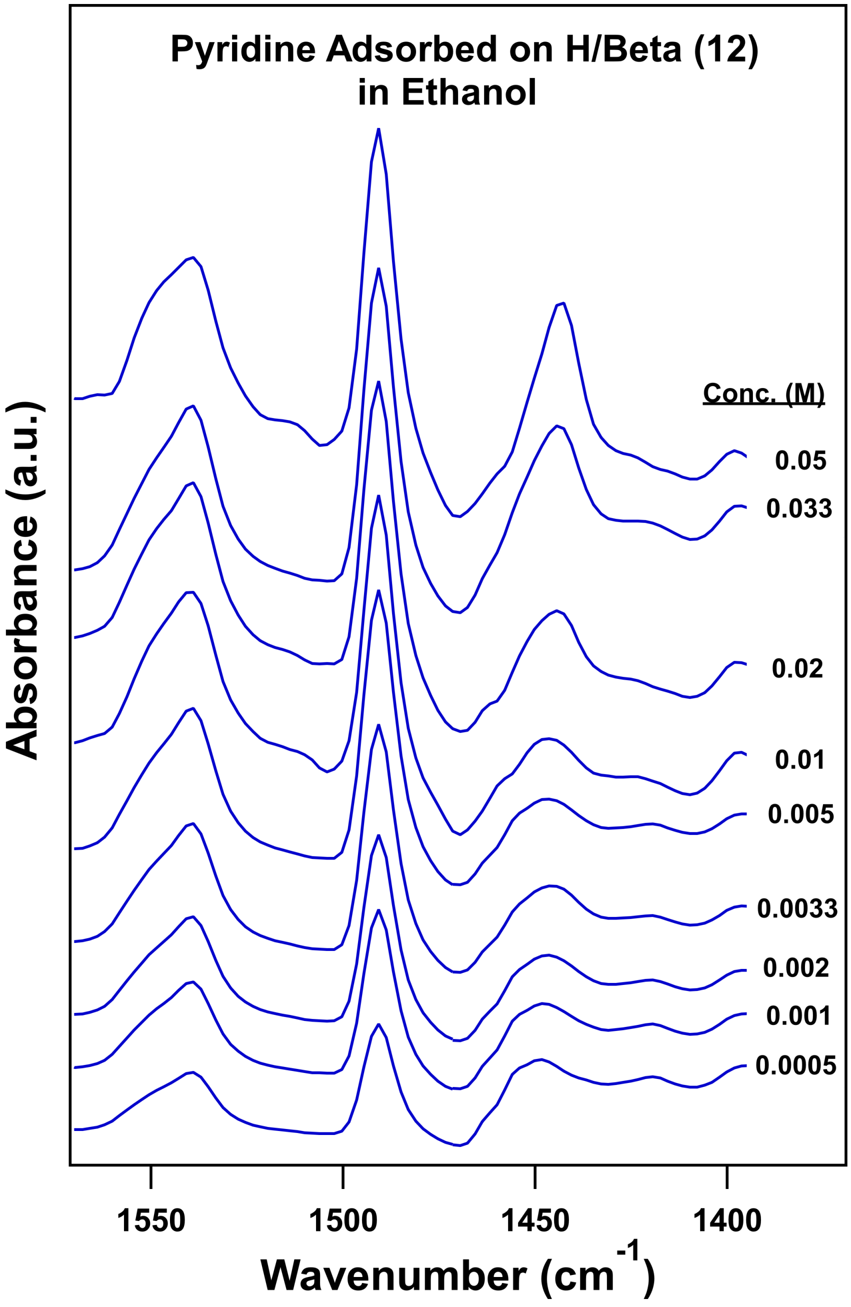

Supplementary Figure 11. ATR-FTIR spectra of pyridine adsorbed on zeolites. ATR-FTIR spectra of pyridine adsorbed on H/Beta (Si/Al = 12) in liquid ethanol at 20 °C. Source data are provided as a Source Data file.

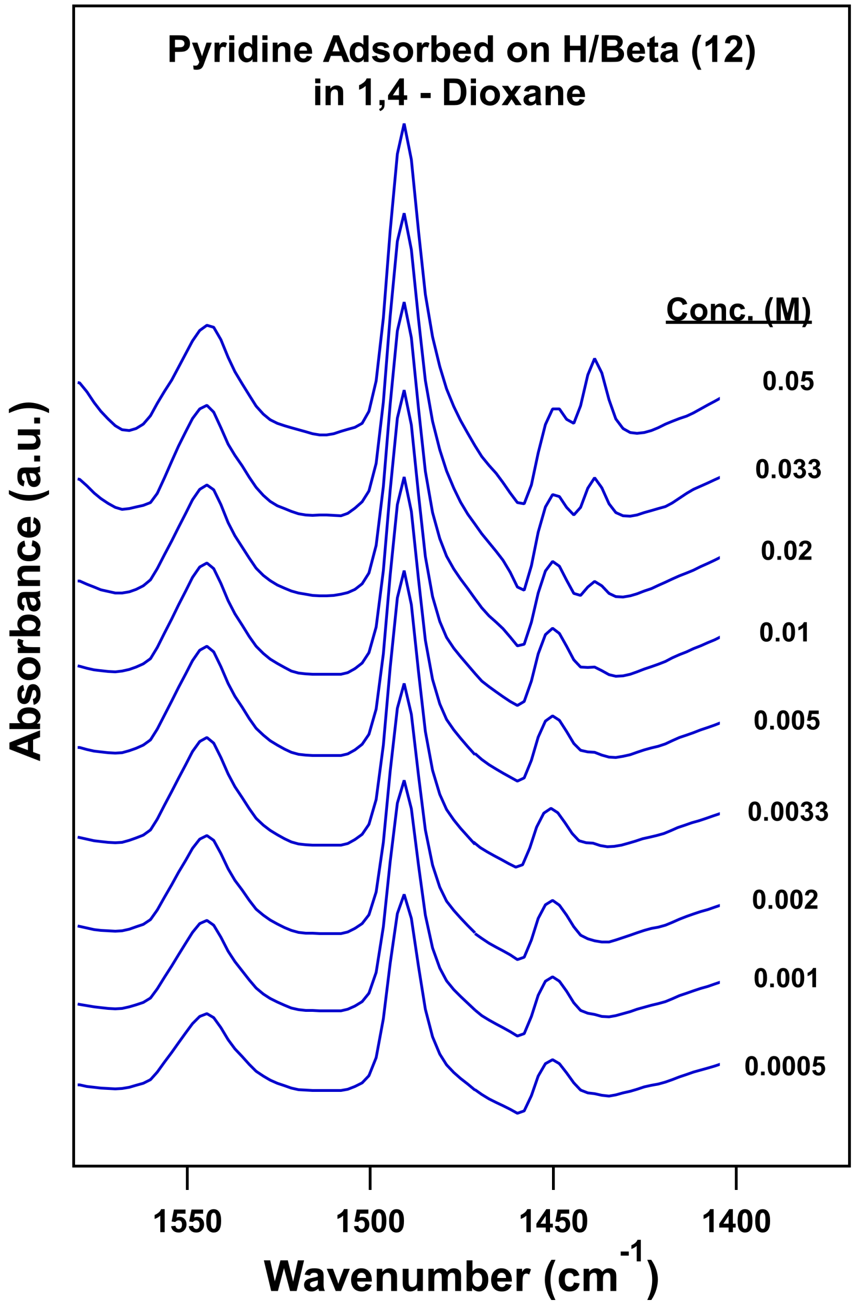

Supplementary Figure 12. ATR-FTIR spectra of pyridine adsorbed on zeolites. ATR-FTIR spectra of pyridine adsorbed on H/Beta (Si/Al = 12) in liquid 1,4-Dioxane at 20 °C. Source data are provided as a Source Data file.

Supplementary Table 3. Energies of the NC (neutral complex), of the IP (ion pair) and of proton transfer (PTE) for pyridine adsorption in H-Beta zeolite in different solvents; PTE calculated from E(IP) – E(NC).

System	Active site	E(NC)	E(IP)	PTE
H/Beta	Al-OH	-1605.66	-1606.32	-0.67
H/Beta + 3 dioxane	Al-OH	-1842.51	-1843.36	-0.85
H/Beta + 6 acetonitrile	Al-OH	-1827.88	-1830.03	-0.93
	$H(CH_3CN)_2^+$	-1828.74	-1830.03	-1.29
H/Beta + 7 acetonitrile	$H(CH_3CN)_2^+$	-1866.28	-1867.36	-1.08
H/Beta + 8 acetonitrile	$H(CH_3CN)_2^+$	-1903.52	-1904.35	-0.83
H/Beta + 9 acetonitrile	Al-OH	-1941.25	-1941.71	-0.46
	$H(CH_3CN)_2^+$	-1941.08	-1941.71	-0.63
H/Beta + 10 acetonitrile	Al-OH	-1978.71	-1979.22	-0.51
	$H(CH_3CN)_2^+$	-1978.46	-1979.16	-0.70
H/Beta + 28 water	Al-OH	-2019.84	-2021.23	-1.39
	$H(H_2O)_n^+$	-2020.80	-2021.23	-0.43

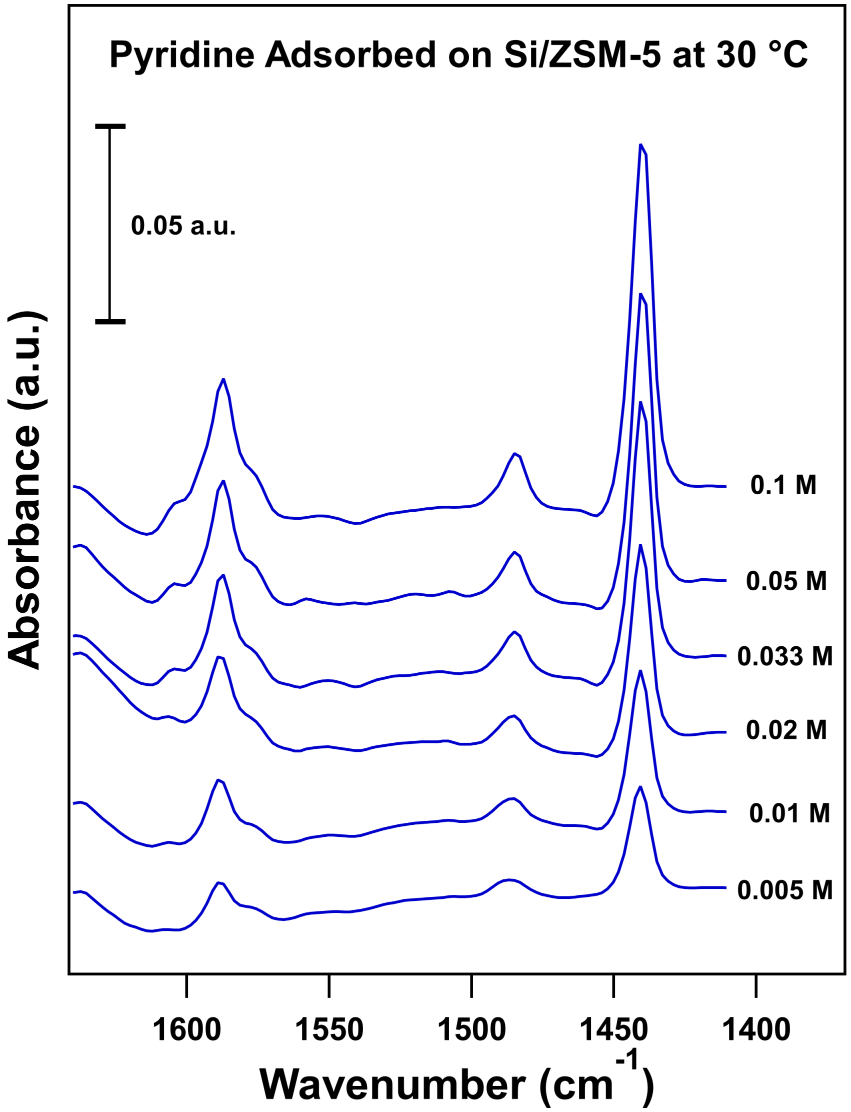

Supplementary Figure 13. ATR-FTIR spectra of pyridine adsorbed on zeolites. ATR-FTIR spectra of pyridine adsorbed on Si/ZSM-5 in liquid water at 30 °C. Source data are provided as a Source Data file.

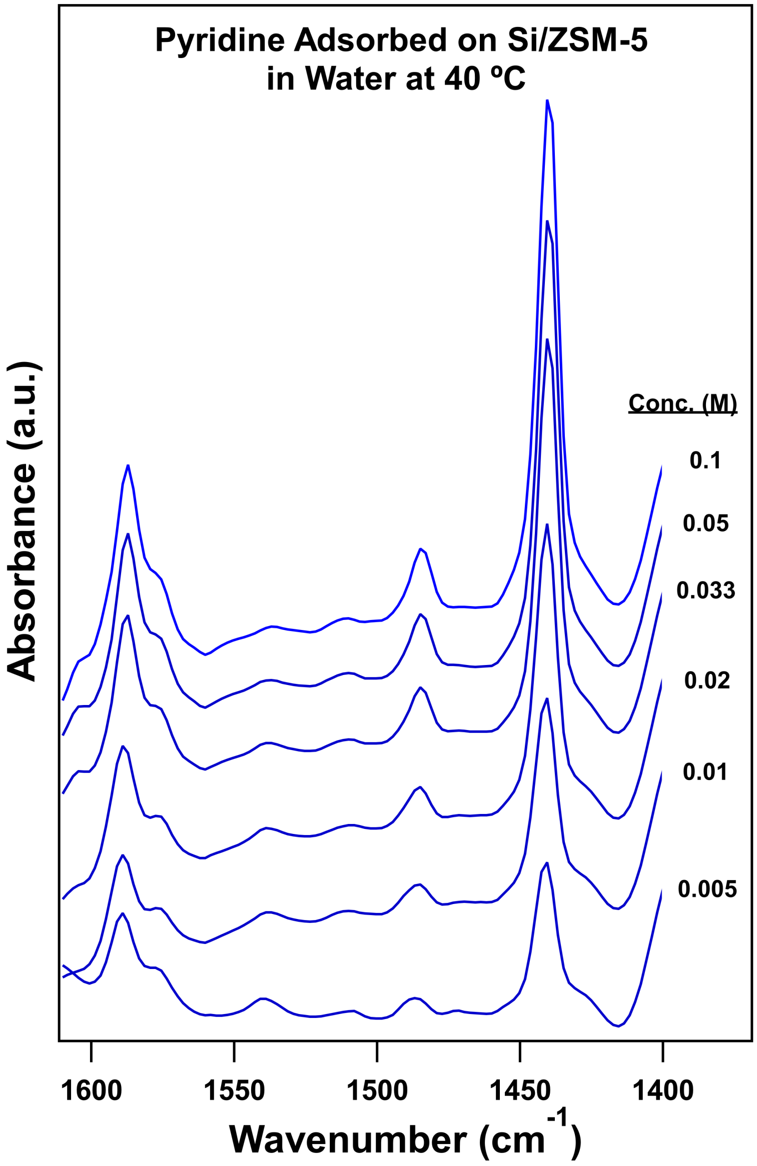

Supplementary Figure 14. ATR-FTIR spectra of pyridine adsorbed on zeolites. ATR-FTIR spectra of pyridine adsorbed on Si/ZSM-5 in liquid water at 40 °C. Source data are provided as a Source Data file.

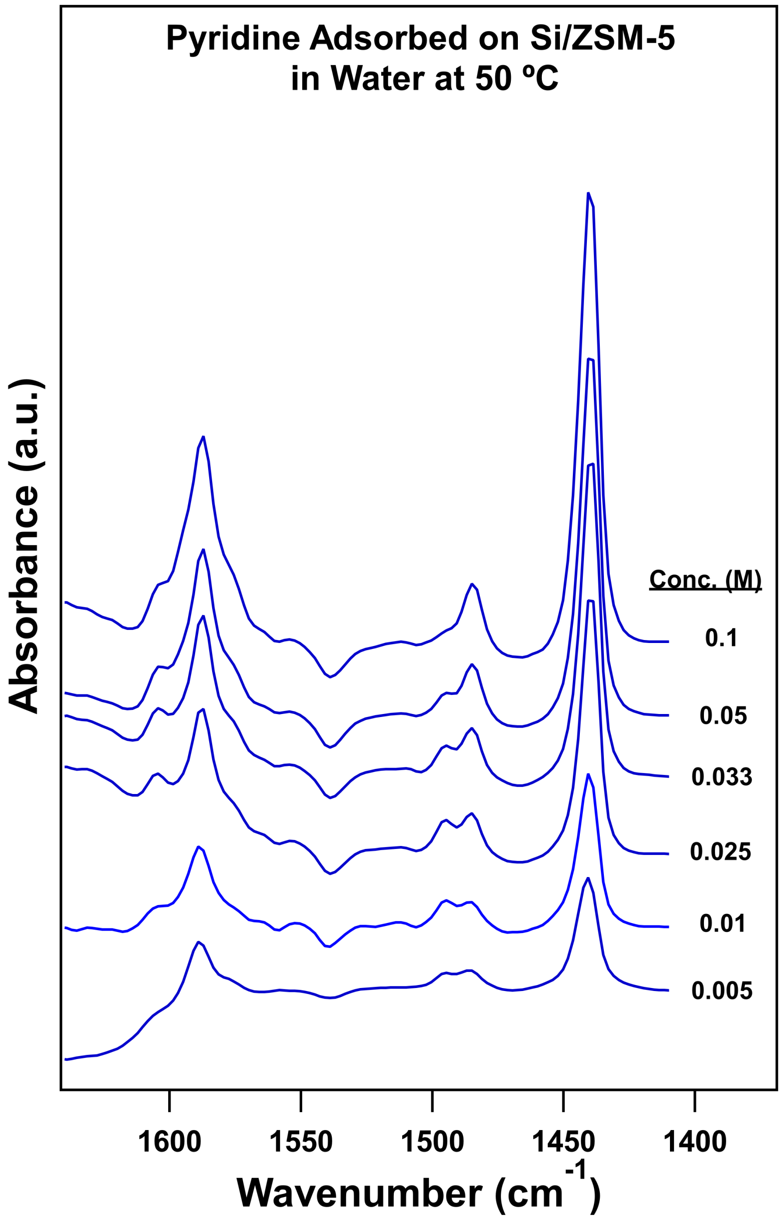

Supplementary Figure 15. ATR-FTIR spectra of pyridine adsorbed on zeolites. ATR-FTIR spectra of pyridine adsorbed on Si/ZSM-5 in liquid water at 50 °C. Source data are provided as a Source Data file.

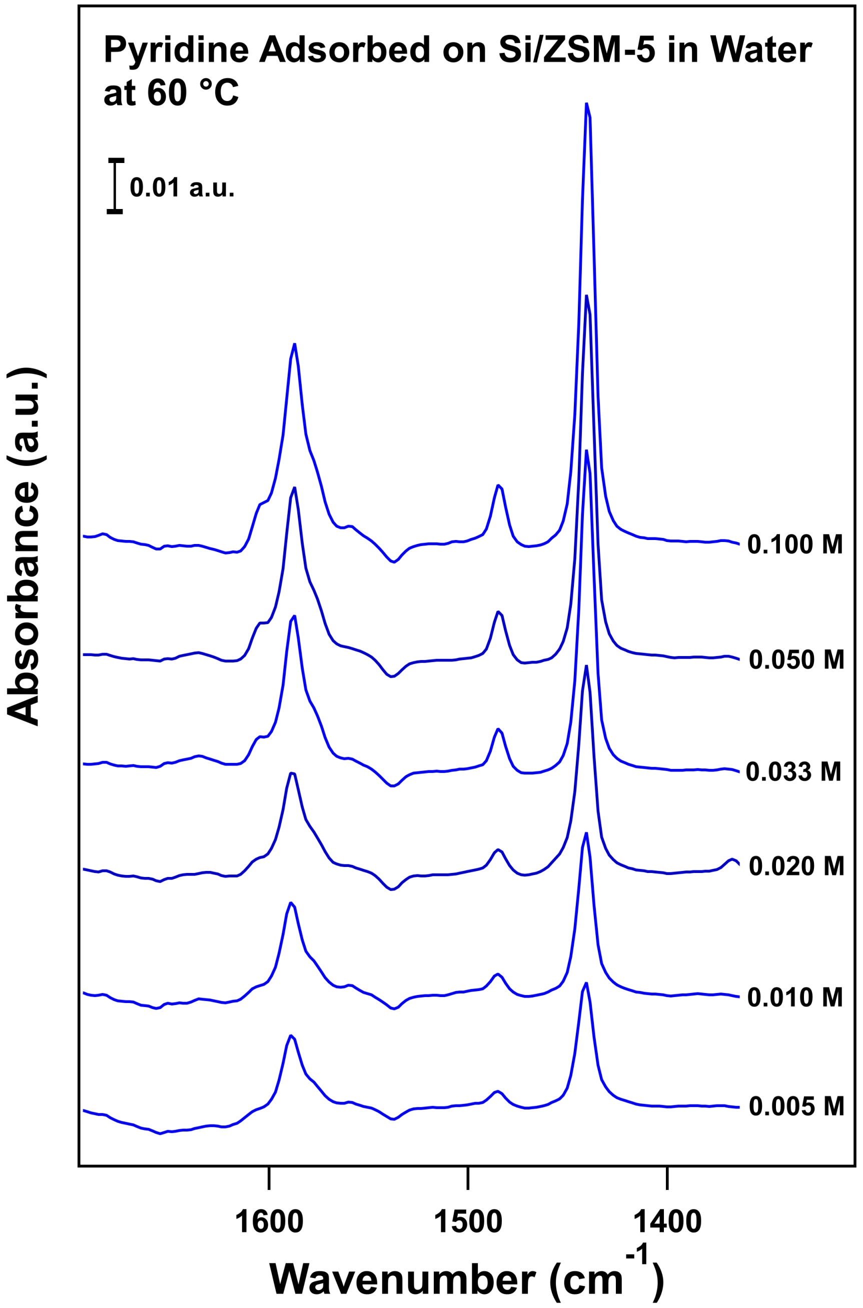

Supplementary Figure 16. ATR-FTIR spectra of pyridine adsorbed on zeolites. ATR-FTIR spectra of pyridine adsorbed on Si/ZSM-5 in liquid water at 60 °C. Source data are provided as a Source Data file.

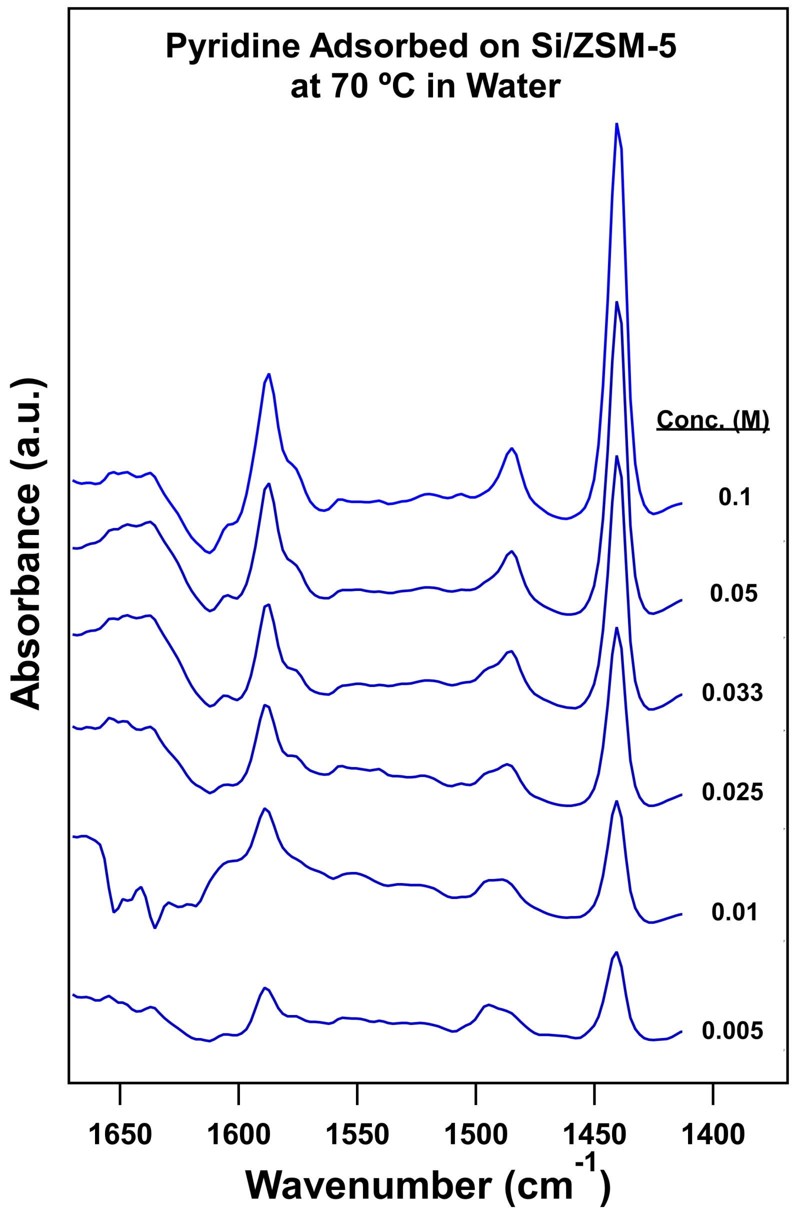

Supplementary Figure 17. ATR-FTIR spectra of pyridine adsorbed on zeolites. ATR-FTIR spectra of pyridine adsorbed on Si/ZSM-5 in liquid water at 70 °C. Source data are provided as a Source Data file.

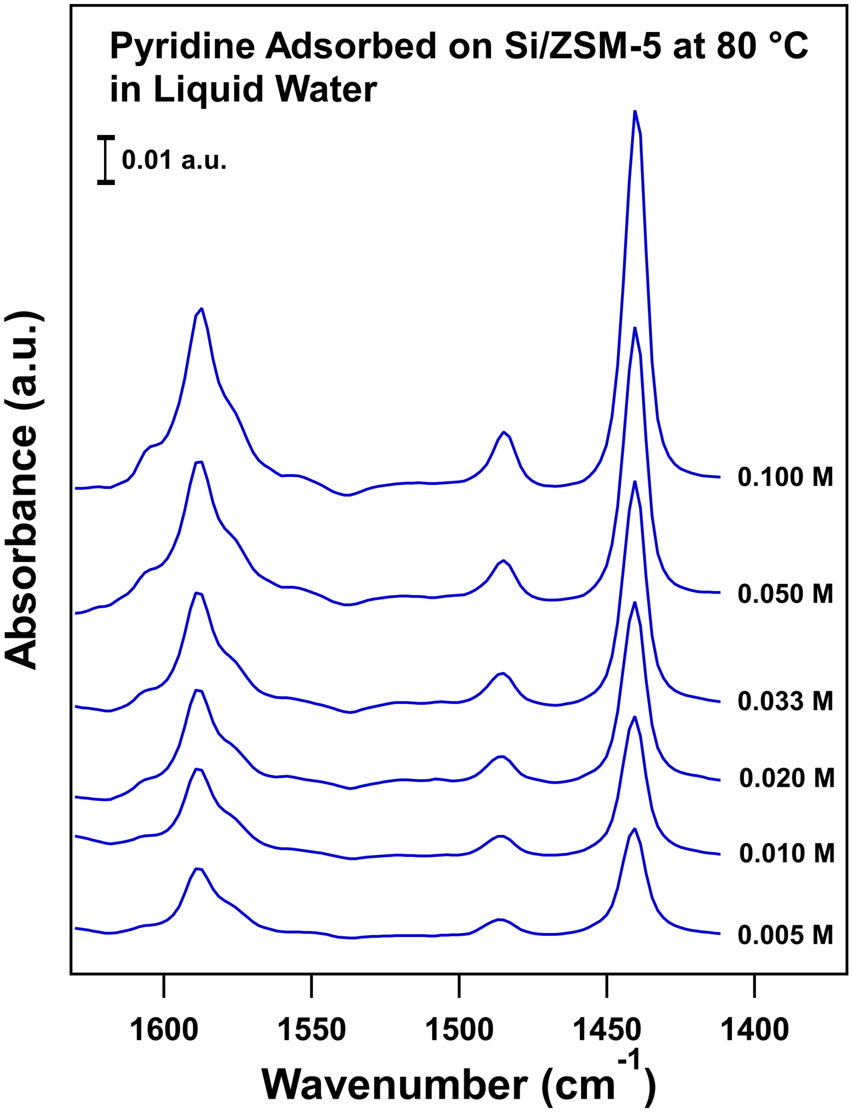

Supplementary Figure 18. ATR-FTIR spectra of pyridine adsorbed on zeolites. ATR-FTIR spectra of pyridine adsorbed on Si/ZSM-5 in liquid water at 80 °C. Source data are provided as a Source Data file.

Supplementary Table 4. Nitrogen adsorption micropore volumes via t-plot method and ^{29}Si NMR results of zeolite samples in this work

Zeolite Sample (nominal Si/Al)	Si/Al Ratio Via ^{29}Si NMR	Micropore Volume (mL g^{-1})
H/ZSM-5 (11.5)	12	0.12
H/ZSM-5 (40)	36	0.13
Si/ZSM-5	N/A	0.13
Si/Beta (hydrophobic)	N/A	0.18
Si/Beta (hydrophilic)	N/A	0.17
H/Beta (12)	12	0.18
H/Beta (150)	N/A	0.15
H/Y (3)	3.6	0.31
H/Y (15)	12	0.27

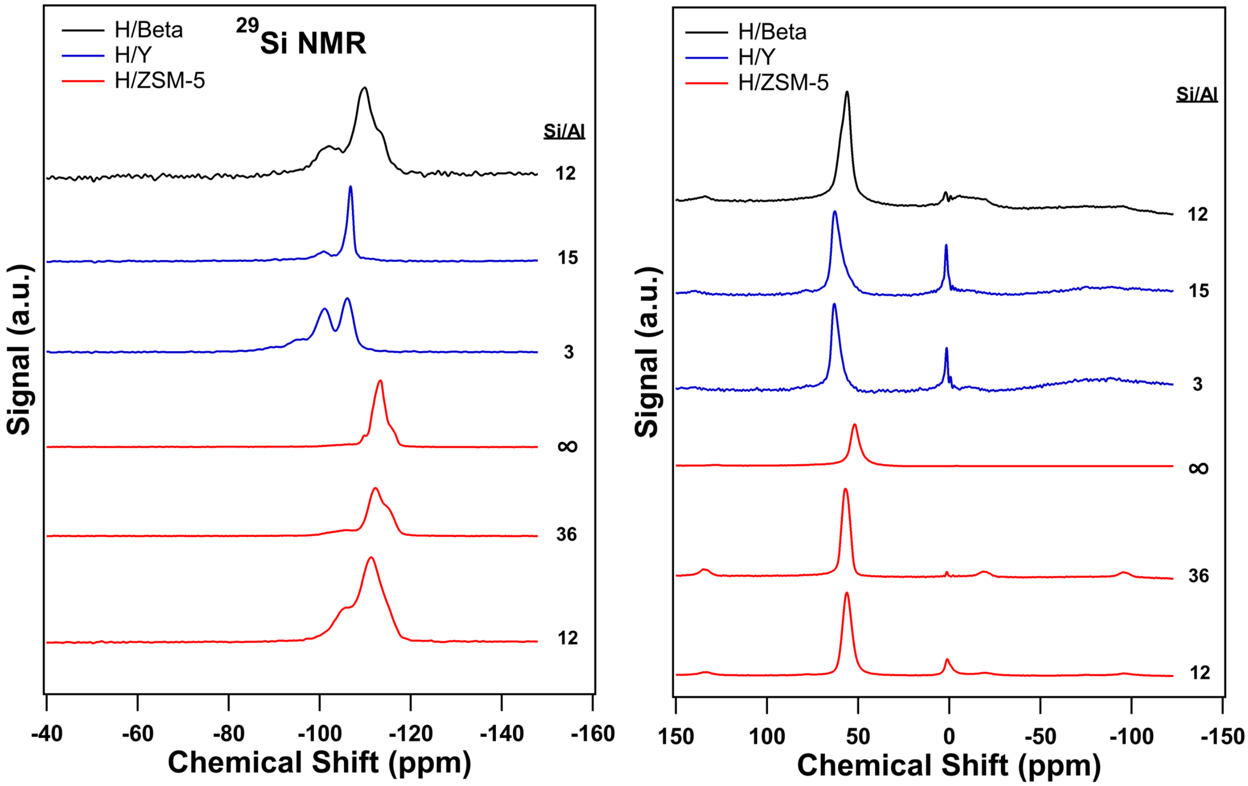

Supplementary Figure 19. Solid state NMR spectra of zeolite samples investigated in this work. ^{29}Si NMR (left) and ^{27}Al NMR (right) of zeolite samples in this work.

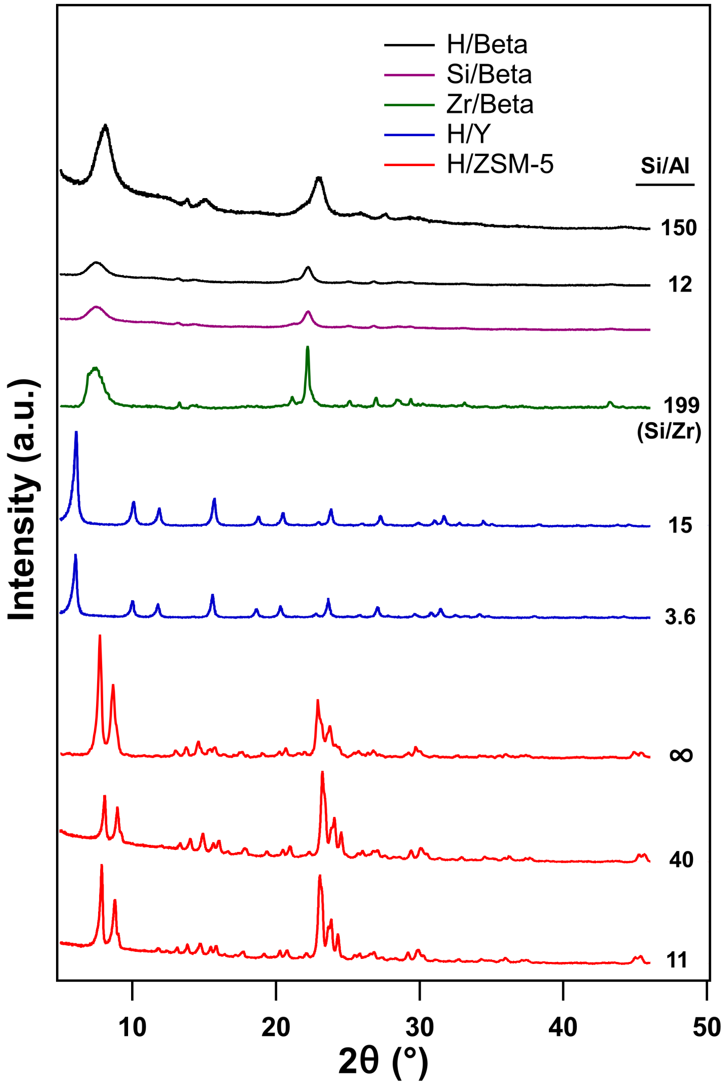

Supplementary Figure 20. X-Ray Diffraction (XRD) patterns of zeolite samples investigated in this work.

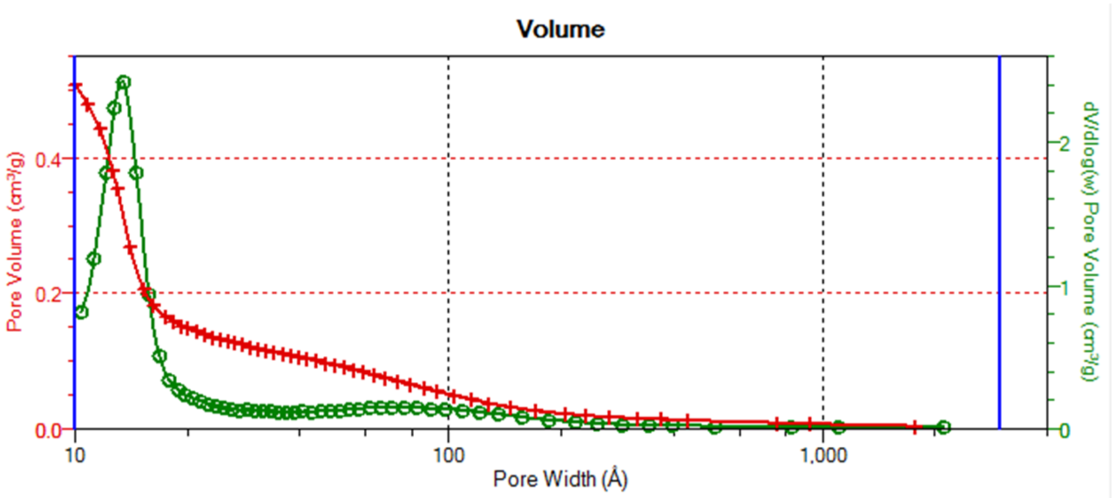

Supplementary Figure 21. BJH isotherm displaying pore width and volume distribution of Si/MCM-41 (1.5 nm).

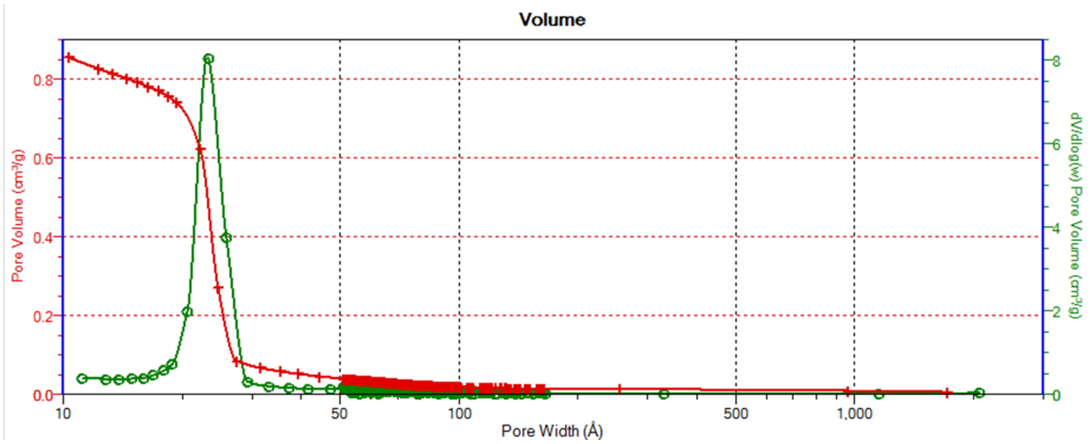

Supplementary Figure 22. BJH isotherm displaying pore width and volume distribution of Si/MCM-41 (2.5 nm).

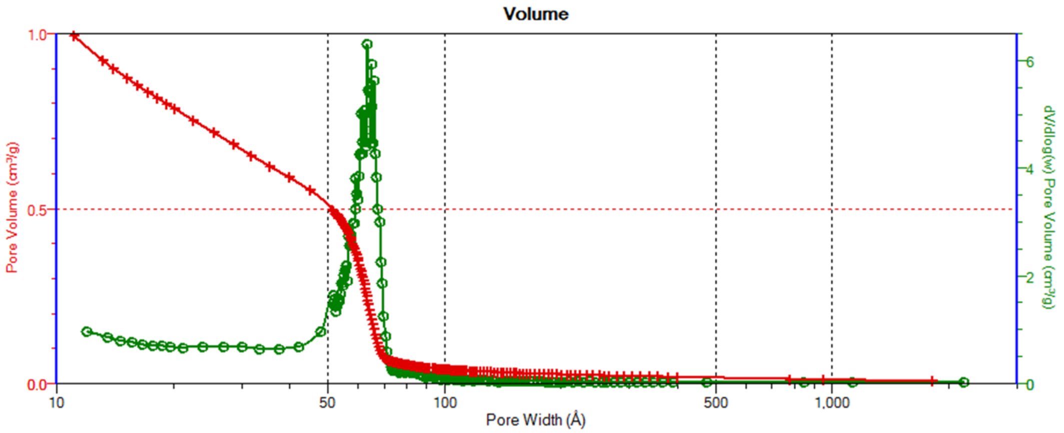

Supplementary Figure 23. BJH isotherm displaying pore width and volume distribution of Si/SBA-15 (6.5 nm).

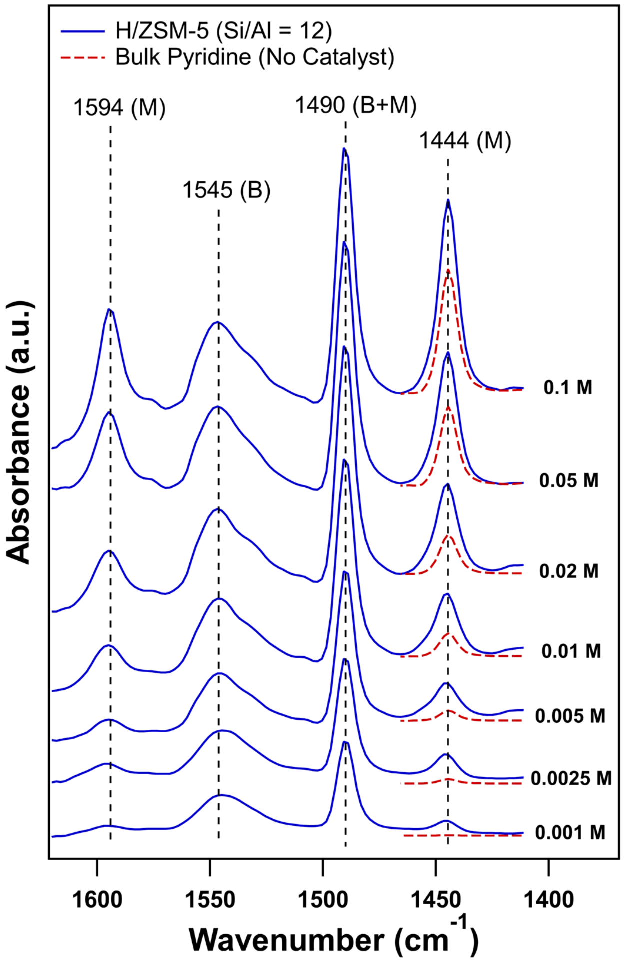

Supplementary Figure 24. ATR-FTIR spectra of pyridine in H/ZSM-5. ATR-FTIR spectra of increasing liquid pyridine concentrations in water on H/ZSM-5 (Si/Al = 12) at 20 °C (blue). The external, liquid pyridine concentration is listed on the right. The liquid pyridine IR signal in an experiment devoid of catalyst is in red (dashed). Source data are provided as a Source Data file.

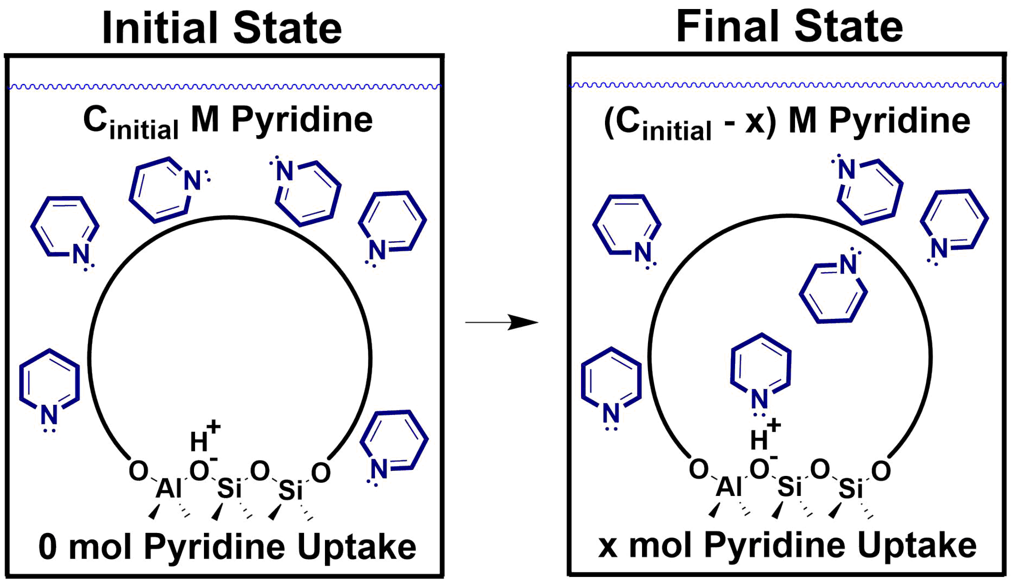

Supplementary Figure 25. Scheme showing initial and final states of pyridine in a solution exposed to a porous material. Phase equilibria experiment to guide quantification of adsorbed pyridine and the fraction of IR signal contacting catalyst particles in ATR-FTIR.

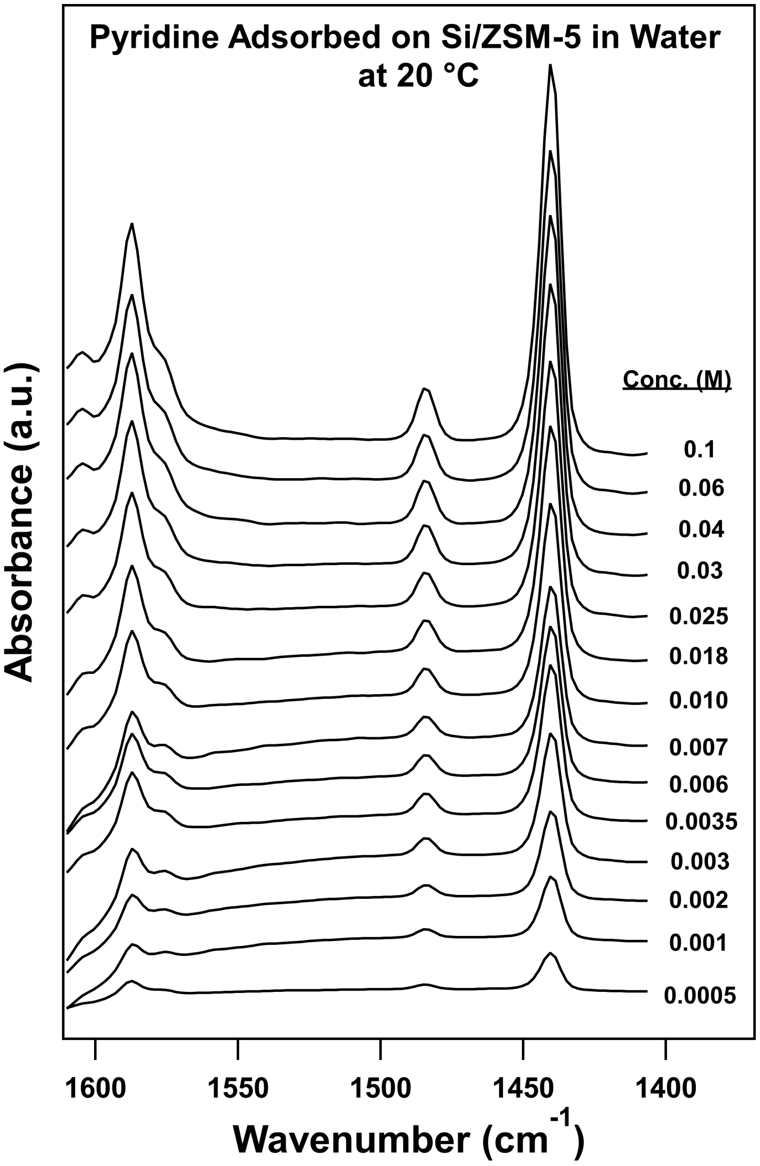

Supplementary Figure 26. ATR-FTIR spectra of pyridine adsorbed on zeolites. ATR-FTIR spectra of pyridine adsorbed on Si/ZSM-5 in liquid water at 20 °C. Source data are provided as a Source Data file.

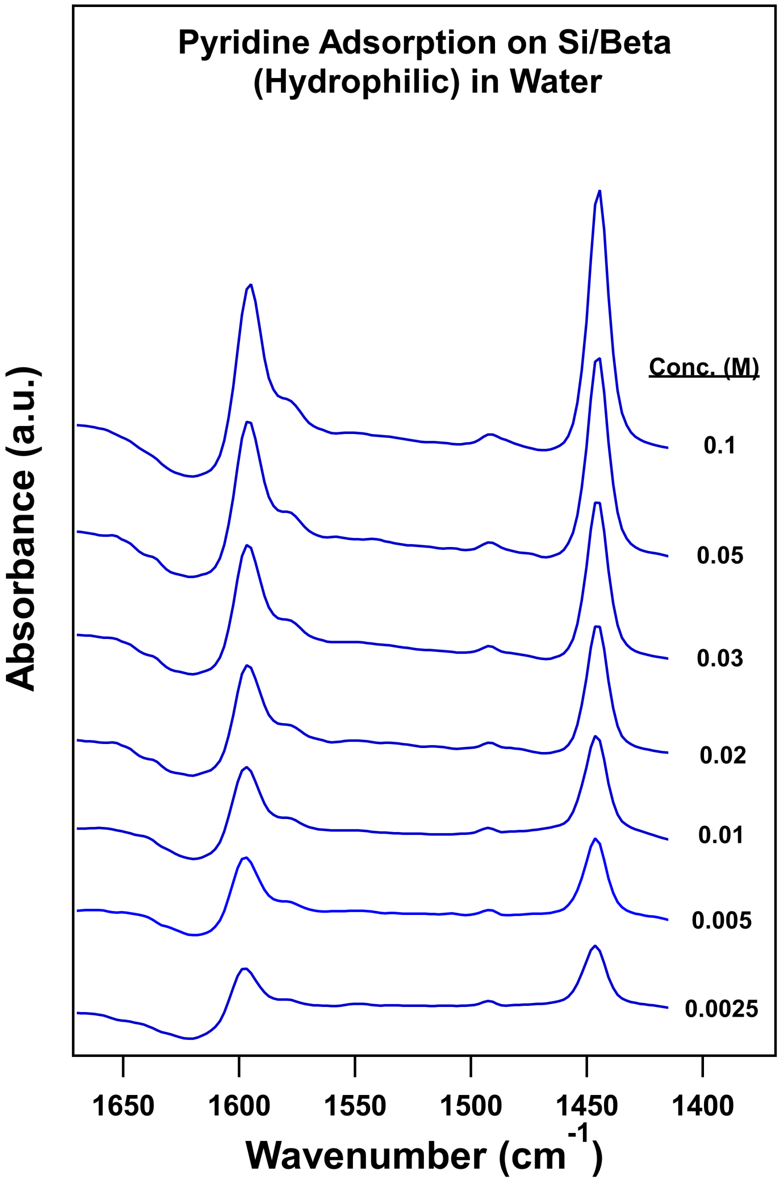

Supplementary Figure 27. ATR-FTIR spectra of pyridine adsorbed on zeolites. ATR-FTIR spectra of pyridine adsorbed on Si/Beta in liquid water at 20 °C. Source data are provided as a Source Data file.

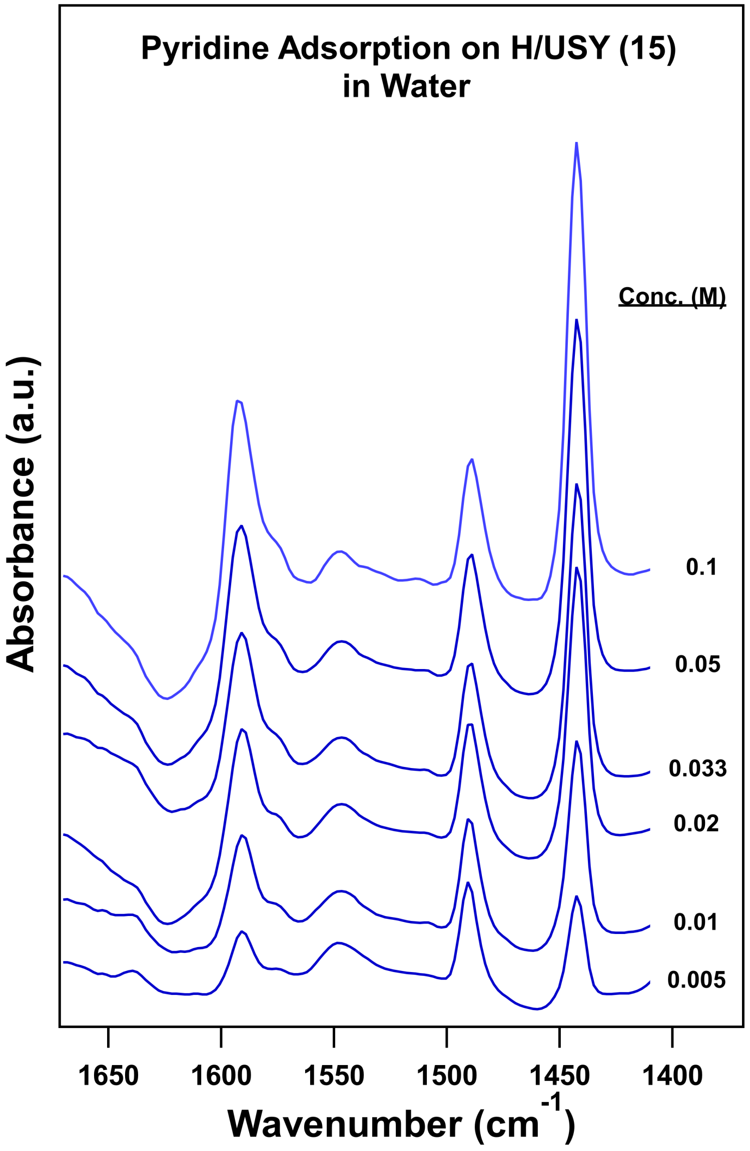

Supplementary Figure 28. ATR-FTIR spectra of pyridine adsorbed on zeolites. ATR-FTIR spectra of pyridine adsorbed on H/Y (Si/Al = 15) in liquid water at 20 °C. Source data are provided as a Source Data file.

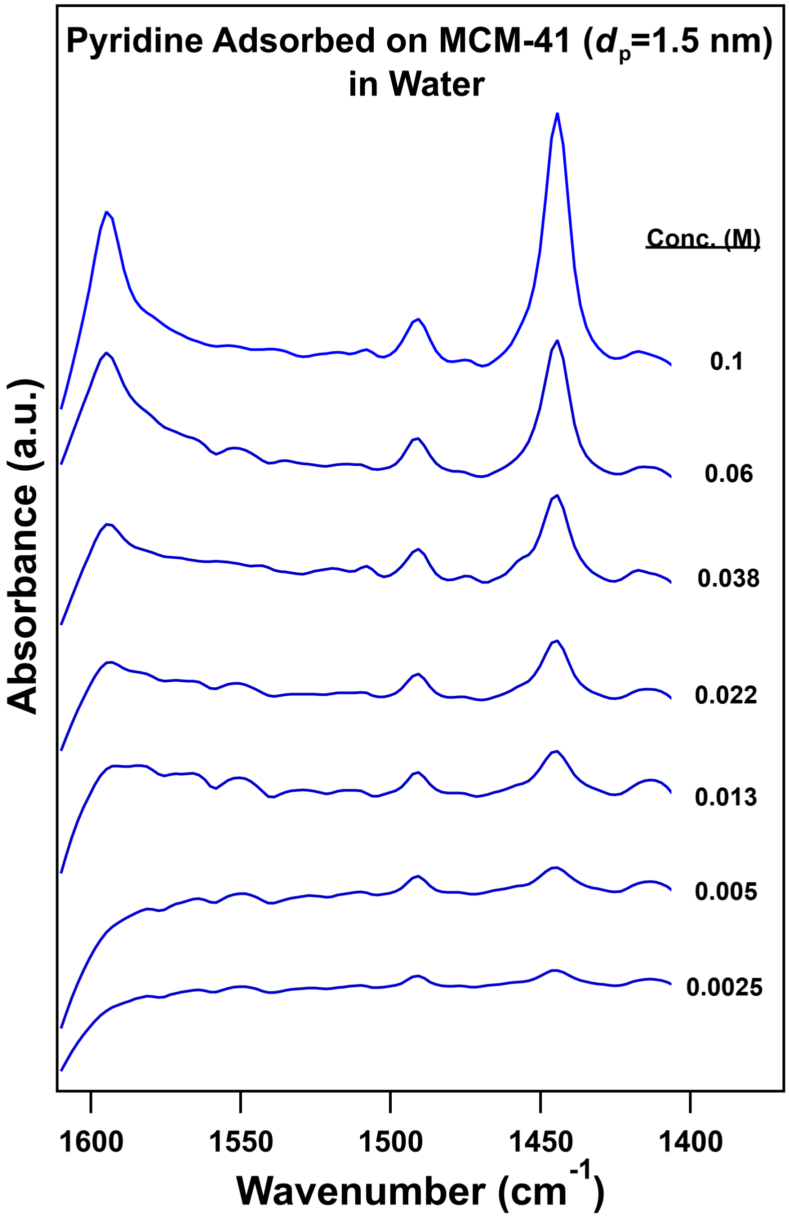

Supplementary Figure 29. ATR-FTIR spectra of pyridine adsorbed on zeolites. ATR-FTIR spectra of pyridine adsorbed on Si/MCM-41 (average pore diameter = 1.5 nm) in liquid water at 20 °C. Source data are provided as a Source Data file.

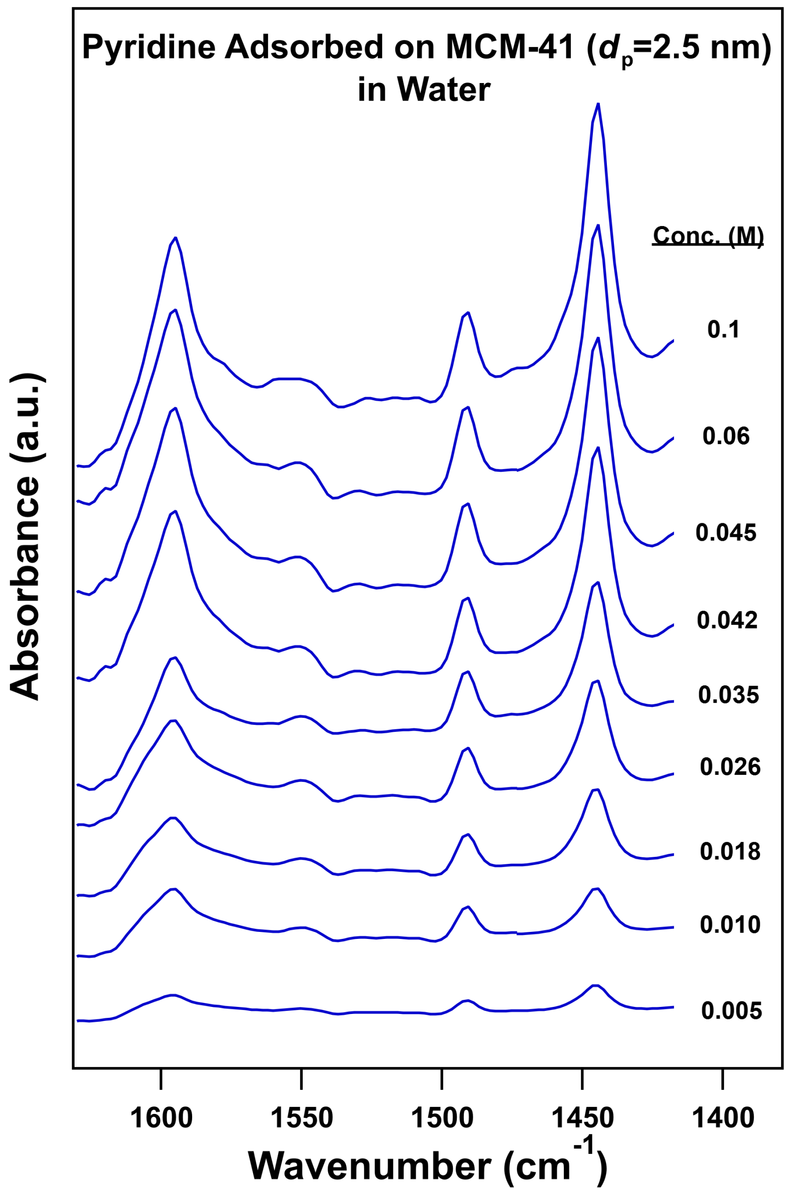

Supplementary Figure 30. ATR-FTIR spectra of pyridine adsorbed on zeolites. ATR-FTIR spectra of pyridine adsorbed on Si/MCM-41 (average pore diameter = 2.5 nm) in liquid water at 20 °C. Source data are provided as a Source Data file.

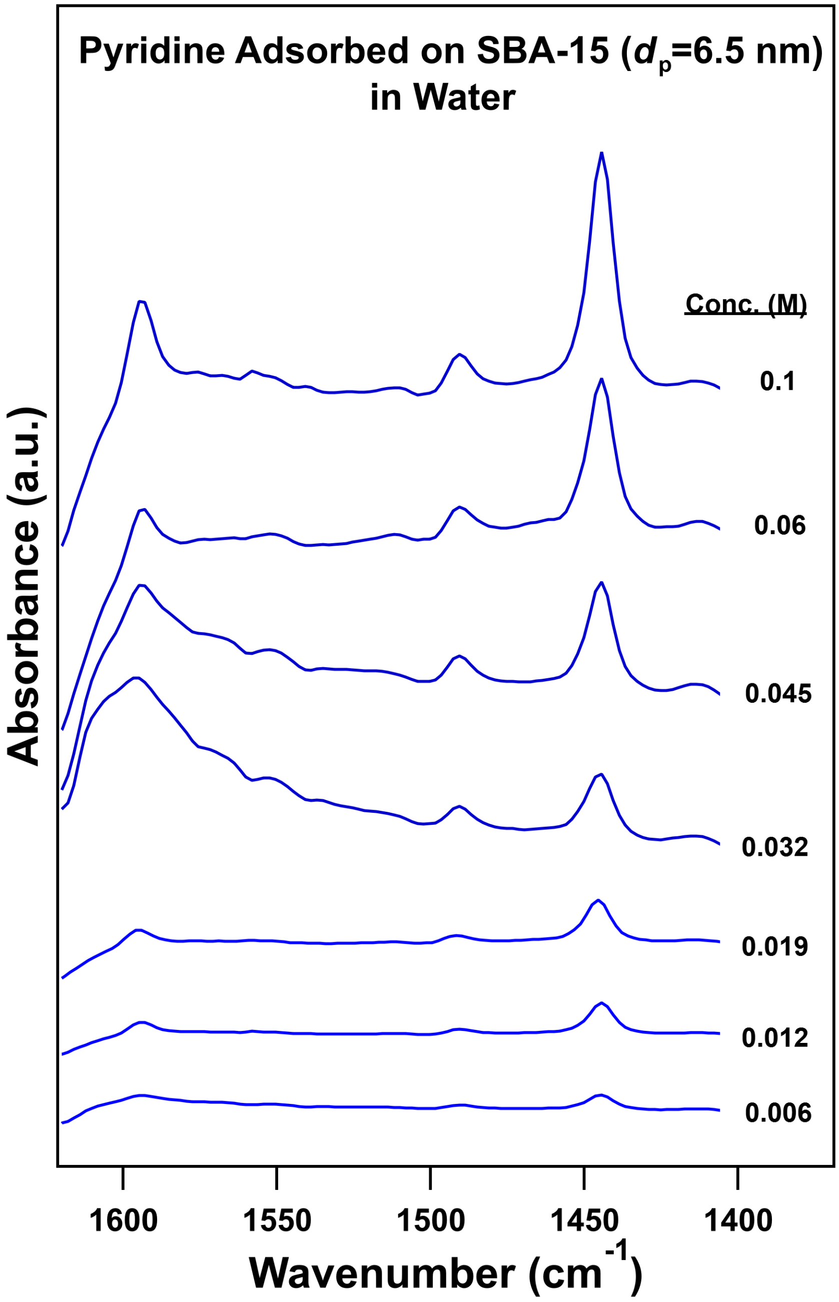

Supplementary Figure 31. ATR-FTIR spectra of pyridine adsorbed on zeolites. ATR-FTIR spectra of pyridine adsorbed on Si/SBA-15 (average pore diameter = 6.5 nm) in liquid water at 20 °C. Source data are provided as a Source Data file.

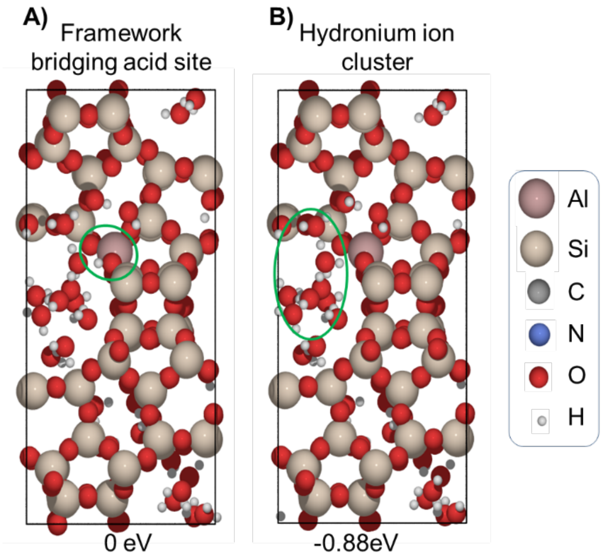

Supplementary Figure 32. DFT optimized H/Beta structure with the proton. On the bridge site (A) and on a water molecule (specific acid site, (B)). Acid sites shown in green circles. Water loading, 28 molecules per unit cell.

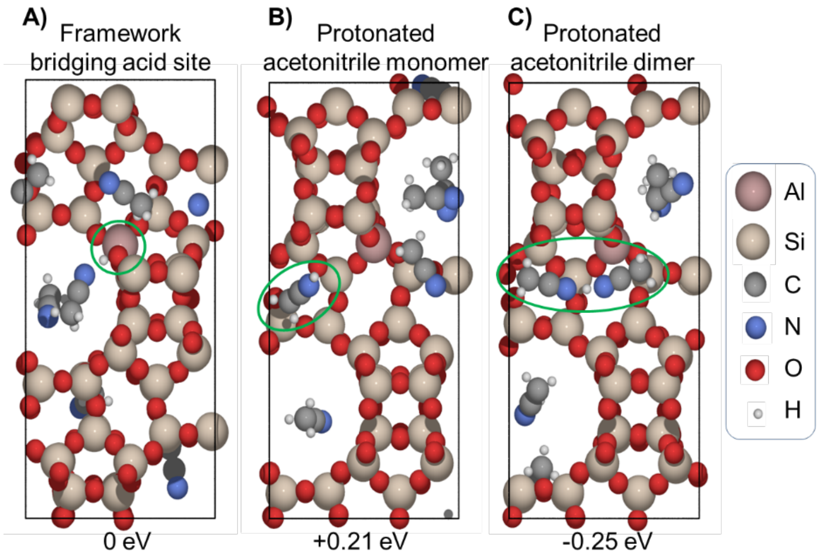

Supplementary Figure 33. DFT optimized structures with 6 acetonitrile molecules per unit cell. A) proton at the bridge site; B) acetonitrile specific acid site with proton coordinated to a single solvent molecule; C) acetonitrile specific acid site with proton stabilized by two coordinated solvent molecules. Acid sites shown in green circles. Relative energies of the three structures are shown under each structure.

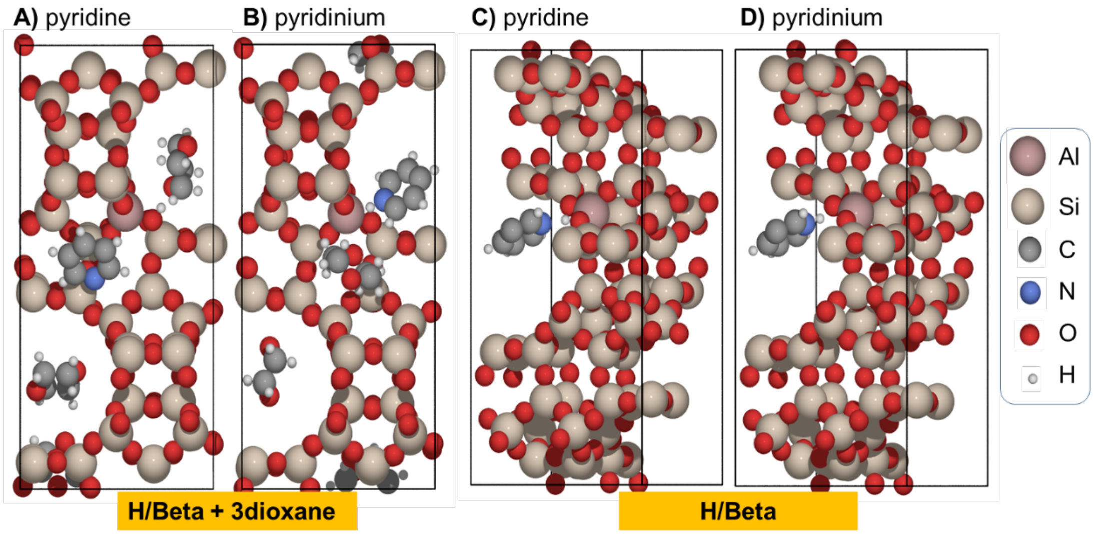

Supplementary Figure 34. DFT optimized structures of pyridine and pyridinium in H/Beta. With 3 dioxane molecules per unit cell (A, B) and in dry H/Beta (C, D).

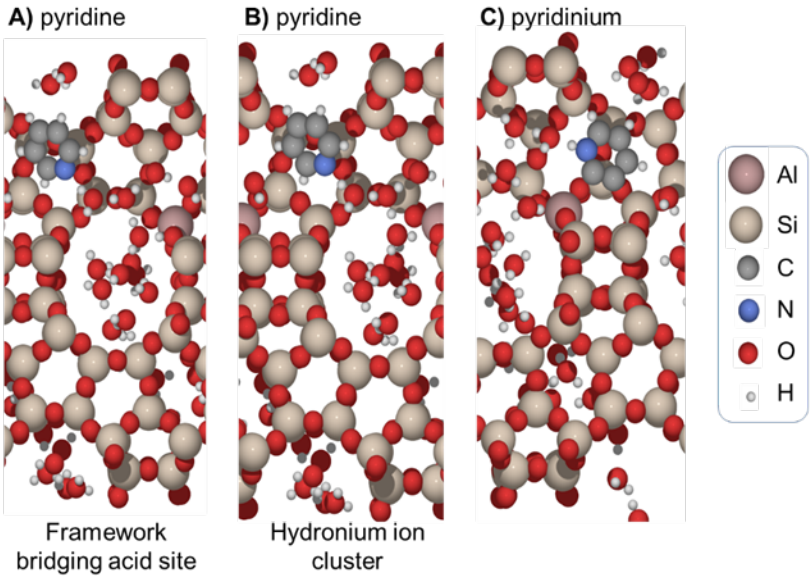

Supplementary Figure 35. DFT optimized structures of H/Beta with water molecules and different forms of the probe molecule pyridine. Pyridine (A, B) and pyridinium (C) in H/Beta with 28 water molecules per unit cell. (A) Proton at Al-O-Si bridge site; (B) proton at specific acid site; (C) protonated pyridine.

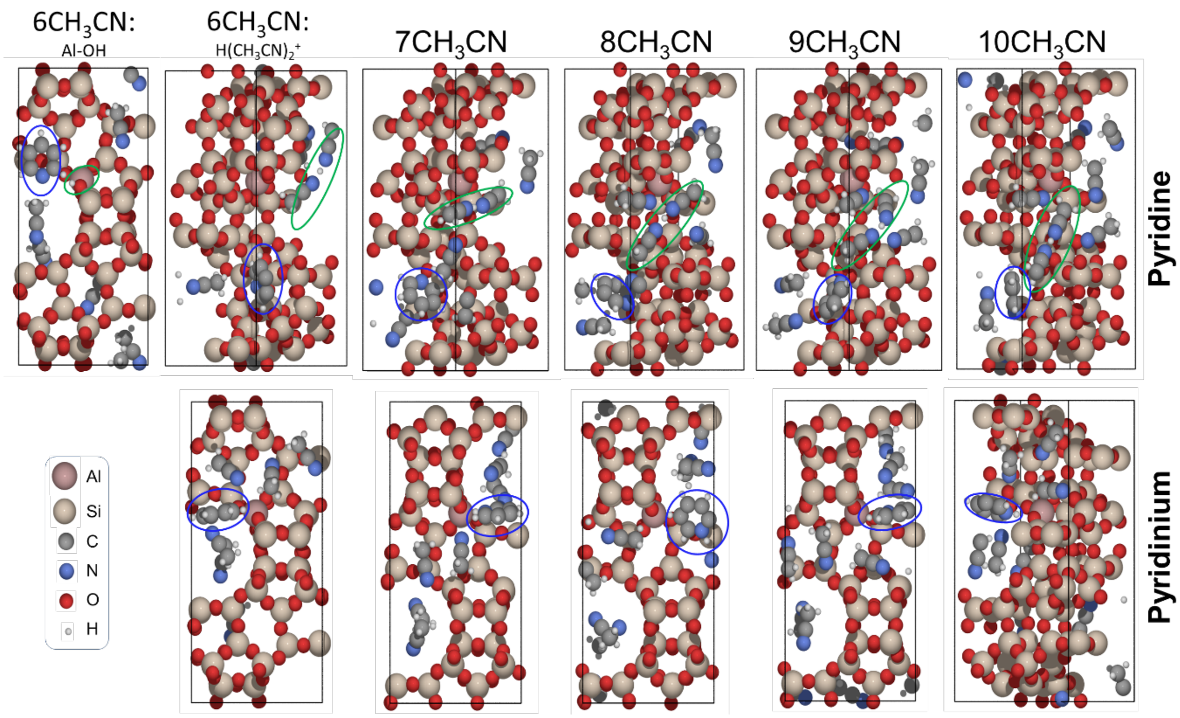

Supplementary Figure 36. DFT optimized structures of H/Beta with different forms of the probe molecule pyridine. Pyridine (top panel) and pyridinium (bottom panel) in H/Beta with 6-10 acetonitrile molecules per unit cell. Acid sites are shown in green circles, while pyridine or pyridinium are shown in blue circles.

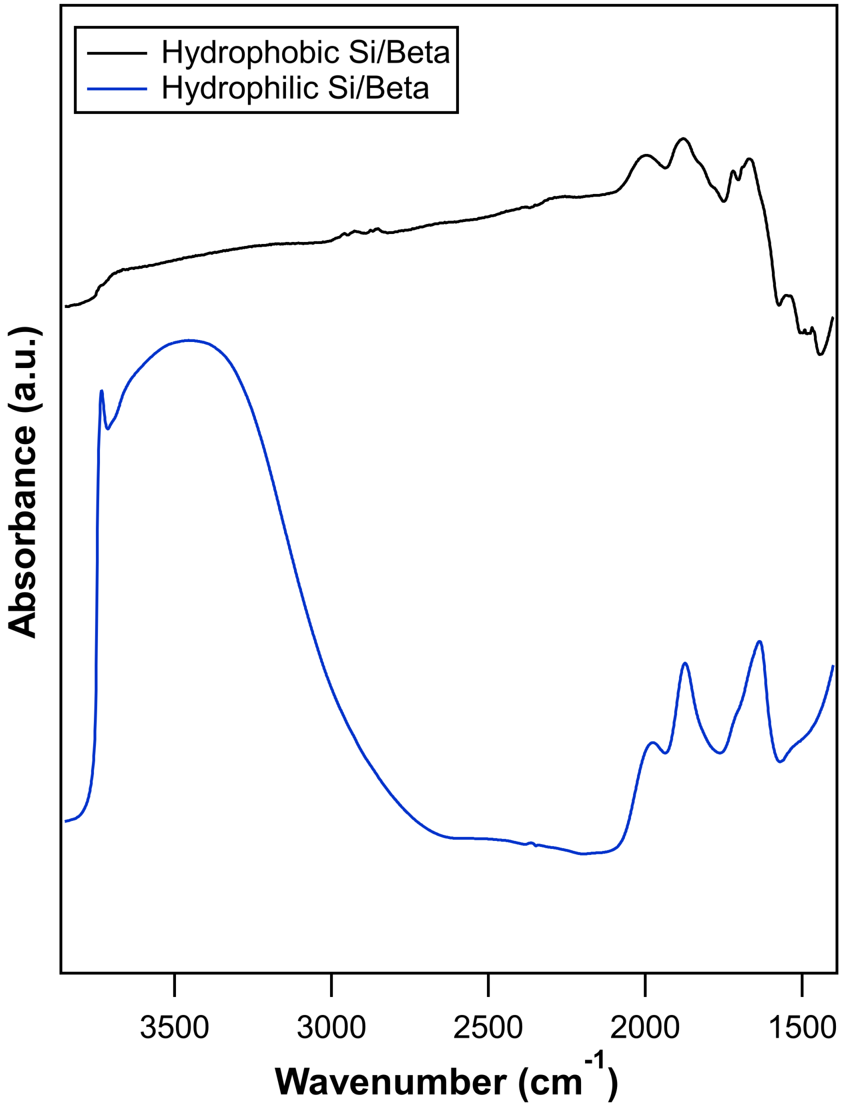

Supplementary Figure 37. Transmission FTIR spectra of zeolites. Vacuum FTIR spectra of the hydrophilic and hydrophobic Si/Beta samples used in this work at 20 °C. Source data are provided as a Source Data file.

Supplementary Discussion

For all the microporous and mesoporous materials employed in this work, the ΔG_{ads} value is most extreme for the smallest pore sizes at the dilute limit. The corresponding changes in free energy of transfer are calculated for the adsorption isotherms in Figure 2A using the relative concentrations of pyridine in the external liquid and the pore phase (Supplementary Equation 1

$$(1) \quad \Delta G_{\mathrm{ads}} = \mu_{\mathrm{Z}}^{*} - \mu_{\mathrm{L}}^{*} = -RT\ln\left(\frac{C_{\mathrm{Z}}}{C_{\mathrm{L}}}\right)$$

and Supplementary Figure 1). Note that in Supplementary Equation 1, two different definitions of the pore-phase concentration are possible. While the adsorption isotherms in manuscript Figure 2A use the sample pore volume, ΔG_{ads} values in Supplementary Figure 1 depend upon either using solely the pore volume to calculate pore-phase concentrations (solid lines) or the volume occupied by both the pores and the framework itself, as would be representative of a unit cell of the material (dashed lines). ΔG_{ads} values based on both definitions can be found in Supplementary Figure 1. For the siliceous Beta and ZSM-5 samples, ΔG_{ads} values range from roughly −15 kJ mol^{-1} at the dilute limit to −9 kJ mol^{-1} by the concentrated end of the isotherm at 0.1 M. Adsorption becomes less favorable, presumably driven by the increasing entropic loss with less free volume, as the pore becomes increasingly saturated. The adsorption isotherms in Figure 2A also allow for estimates of fundamental thermodynamic descriptors of the pore phase, including standard-state chemical potentials ($\mu_{\mathrm{Z,H}}^{\circ}$) and activity coefficients ($\gamma_{\mathrm{Z,H}}$) . The standard-state chemical potentials and activity coefficients in Table 1 and Figure 5 are based on deviations from Henry's law (subscript H) and are calculated by equating the chemical potential of pyridine in the external liquid to that of the zeolite pore (see discussion of Equation 16 for details). The difference in standard-state chemical potentials between the zeolite phase and the liquid phase (water) is roughly −15 kJ mol^{-1} for Beta and ZSM-5 zeolites and roughly −13 kJ mol^{-1} for USY (Supplementary Table 2). These

estimates are of a similar magnitude to the difference between liquid phase and vapor phase standard-state chemical potentials (≈ -14 kJ mol^{-1}), suggesting that pore-phase confinement of pyridine into zeolite pores from liquid water has a similar thermodynamic driving force as ideal-gas pyridine "dissolving" into liquid water. This strong affinity for the pore phase results in a roughly Langmuir adsorption shape in water for Si/ZSM-5 and Si/Beta samples in manuscript Figure 2A. Note however, that the intensity of the chemical potential difference drops for USY, where increasing pore diameter decreases the adsorption strength from the liquid phase. The same phenomenon is observed for the mesoporous materials in Supplementary Figure 1, where negative ΔG_{ads} values are due to stabilizing interactions with the silica surface. These stabilizing interactions with silica in the mesoporous materials are quite weak and become weaker with increasing pore diameter as discussed in the context of Figure 2C. Based on the Henry's law definition, the activity coefficient values in Supplementary Figure 2 are indicative of deviations from dilute solution behavior (infinitely dilute pyridine in the water-saturated pore phase). Note that over the concentration range surveyed in Supplementary Figure 2, the liquid-phase pyridine activity coefficient varies by only 2% from infinite dilution to 0.1 M liquid pyridine based on values estimated with UNIQUAC.[1] Nearly all liquid phase reactions involve non-ideal mixtures where reaction rates are dependent on activities ($\gamma_i \times C_i$), even though most rate expressions are written in terms of concentrations.[2] At equilibrium, chemical potentials are equal across phases, i.e., the liquid and the pore phases. It is important to note that the adsorption isotherms in Figure 2A and the subsequent estimation of standard-state chemical potentials and activity coefficients allow one to fully describe the pore phase environment. This includes the ability to make *quantitative* arguments about the effect of solvent on substrate concentrations in the phase containing the active sites, which could affect active site surface coverage and competitive adsorption, as well as the

substrate energetics and interactions in the pore phase. Decoupling the concentration from the energetic effects is valuable in liquid phase heterogeneous reactions where the number of potential solvent effects is extensive.

For a particular zeolite framework, the total pore-phase concentration of pyridine and pyridinium is similar across all silicon to aluminum (Si/Al) ratios in liquid water. Pyridine adsorption isotherms for several H/ZSM-5 and H/Beta samples with varying Si/Al ratios are shown in Supplementary Figure 3. The fitted lines through the data are the result of regression to optimize the *K*' parameter in a Langmuir isotherm model (Supplementary Equation 2). The observation that

$$(2) \qquad \theta = \frac{K' C_{\mathrm{pyr,L}}}{1 + K' C_{\mathrm{pyr,L}}} \qquad \text{(Ideal Langmuir Isotherm)}$$

the sum of pyridine and pyridinium concentrations is nearly constant across all aluminosilicate zeolite samples is intuitive at the concentrated end of the isotherm (Supplementary Figure 3), which also reflects the quantitative accuracy of the experiment and analytical method employed in this work. However, the greater *K*' parameter for pyridine on BAS compared to MAS (Supplementary Table 1) suggests that near infinite dilution, the total pore-phase pyridinium + pyridine concentration in Al-containing samples exceeds that of purely siliceous samples, which can be attributed to the stronger adsorption and protonation of pyridine on BAS. It is interesting to contrast our observations here with the report by Eckstein et. al., where the saturation loadings for the organic adsorbates decrease with increasing Al substitution, a phenomenon attributed to non-displaceable water-solvated hydronium clusters.[3] Such strong solvated hydronium clusters can, however, clearly transfer the protons in the presence of strong bases used as adsorbates in this work. It is worth noting that the *K*' parameter is an equilibrium constant for an ideal Langmuir isotherm and has units of M^{-1}. At infinite dilution, the Langmuir isotherm model reduces to Henry's law and can be used to calculate thermodynamic properties when scaled by the maximum

pore phase concentration, i.e., $\Delta G_{ads} = -RT\ln(K' \times C_{Z,max})$.[4] At infinite dilution, this expression is equivalent to that of Supplementary Equation 1.

In liquid water, the free energy of transfer for adsorption into Si/Beta samples is only weakly dependent on the zeolite hydrophilicity. The hydrophilic Si/Beta sample in Supplementary Figure 3 is prepared via dealumination of H/Beta (12) with nitric acid and the hydrophobic Si/Beta sample is synthesized via a fluoride-mediated route. Transmission FTIR spectra of the two Si/Beta samples under vacuum qualitatively confirm the hydrophilic/hydrophobic properties of the two samples (Supplementary Figure 32), with the hydrophobic sample exhibiting much weaker bands for adsorbed water at 20 °C. For MAS, the hydrophobic Si/Beta sample results in a greater K' parameter (Supplementary Table 1) than the hydrophilic and Al containing samples, suggesting that pyridine adsorption into the hydrophobic BEA sample is more favorable than the hydrophilic ones. Hydrophobic samples have a lesser affinity for water, and the steeper isotherm is likely the result of the more easily displaced water molecules and/or the lower concentration of pore-phase water molecules prior to introducing pyridine. Five repetitions of the pyridine isotherms in liquid water on the hydrophilic Si/Beta sample show that the average and standard deviation of K' values is 207 ± 19 (Supplementary Figure 4). This suggests the MAS K' values for the hydrophilic Si/Beta and H/Beta samples are the same within statistical uncertainty. This is expected, as H/Beta is also hydrophilic and molecularly adsorbed pyridine experiences a similar environment. However, the hydrophobic Si/Beta K' value is statistically greater than that of the hydrophilic Si/Beta. Despite the large disparity in K' values, the difference between ΔG_{ads} values for the hydrophobic and hydrophilic Si/Beta samples is only 1.6 kJ mol^{-1} (Supplementary Table 1). The difference between the ΔG_{ads} values for the two Si/Beta samples ($\Delta\Delta G_{ads}$) can be estimated by Supplementary Equation 3, knowing that for a common solvent, the ratio of two K' values will be roughly equal

to the ratio of true equilibrium constants: Applying Supplementary Equation 3 to Si/Beta (hydrophilic) and Si/Beta (hydrophobic) results in a $\Delta\Delta G_{ads}$ of 1.4 kJ mol^{-1}, in good agreement with the 1.6 kJ mol^{-1} value based on the estimation of ΔG_{ads} values via Supplementary Equation 1. Thus, the Langmuir assumption is reasonably accurate for MFI and BEA samples (Figure 2). Note

$$(3) \quad \Delta\Delta G_{\text{ads}} \approx -RT\ln\left(\frac{K_2'}{K_1'}\right)$$

that unlike the ΔG_{ads} values in Supplementary Figure 1, any thermodynamic estimates based on the *K*' values, i.e., Supplementary Equation 2, rely upon the assumptions of the Langmuir model. However, the *K*' values are more statistically sensitive than ΔG_{ads} values for distinguishing the adsorption properties of different samples and showcase the sensitivity of the adsorption isotherm technique in this work.

The presence of water similarly affects the BAS of the three H/ZSM-5 and H/Beta samples (Supplementary Figure 3). The *K*' values for MAS and BAS isotherms agree with the current understanding of the strength of zeolite acid sites in a number of ways. The first is that in a given aluminum-containing zeolite, the *K*' value for BAS is greater than that of MAS, suggesting that protonation of the pore-phase base is thermodynamically more favorable. Further, the *K*' values for BAS agree with results from temperature program desorption (TPD) of pyridine under vacuum in our previous work, where under identical conditions the fraction of pyridine desorbed from BAS by 300 °C on the H/ZSM-5 (36), H/ZSM-5 (12), and H/Beta (12) samples was 11 %, 22 %, and 31 %, respectively (Supplementary Table 1).[5] The qualitative agreement between the BAS *K*' values for these zeolite samples and the vacuum phase pyridine TPD results suggest that water does not affect the ranking order of the interaction strength of pyridine with the BAS of the three aluminum-containing zeolite samples in Supplementary Table 1. While the presence of water will reduce a proton's ability to protonate pyridine compared to protonation in vacuum,[5] the zeolite

with the strongest pyridine-BAS interaction in vacuum also exhibits the strongest interaction in the presence of water (H/ZSM-5, Si/Al = 36). Similarly, BAS on H/Beta (Si/Al = 12) exhibit the weakest interaction with pyridine in both vacuum and in water. This suggests that water has a similar effect on the protons in all three zeolite samples.

Concerning the computational calculations, we have investigated solvent effects on the acidity of H/Beta zeolite by computing the PTE from the acid site to the substrate molecule (pyridine) for three of the solvents considered in the experiments, *viz*., 1,4-dioxane, acetonitrile and water. We define the PTE as the difference between the energy of the pyridinium-conjugate base pair and the energy of the pyridine-acid neutral complex, PTE = E(IP) – E(NC). For the calculation of E(IP), the proton is *constrained* on the substrate and the rest of the system is allowed to relax within the pore in the presence or absence of solvent. Without solvent in the zeolite, the protonated substrate molecule is invariably coordinated to the Al-O-Si bridge that donates the proton. In the presence of solvent, the pyridinium ion is coordinated by solvent molecules. For the calculation of E(NC), the proton is *constrained* on the acid site, which, as we will see below, may be either the Al-O-Si bridge or the specific acid (*viz*., protonated solvent molecule). For dioxane solvent, we have included 3 molecules per unit cell; for water, we have considered loading of 28 molecules per unit cell;[6-10] and for acetonitrile we have varied the loading from 6 to 10 molecules per unit cell.

Prior to adding pyridine in the zeolite, we relaxed the solvent-containing systems by running 2-ps-long AIMD trajectories. Both in the case of water and in the case of acetonitrile, the relaxed configurations showed spontaneous proton transfer from the Al-O-Si bridge site to a solvent molecule, forming the specific acids $[(H_2O)_nH]^+$ and $[(CH_3CN)_nH]^+$, respectively. Optimization of these specific acid sites showed that they are more stable than the bridge acid sites

by *ca*. 0.88 and 0.25 eV for water and acetonitrile, respectively (see Supplementary Figures 33 and 34). This is not surprising: whereas the proton affinity of a single water molecule is *ca*. 7 eV, the proton affinity of a water trimer is already equal to that of ammonia,[10] *ca*. 9 eV. Similarly, whereas a single acetonitrile molecule is not able to hold on to a proton (proton transfer from the bridge is endoergic by ca. 0.2 eV), the dimeric $[(CH_3CN)_2H]^+$ cluster is basic enough to abstract the proton of the Al-O-Si bridge (proton transfer from the bridge is exoergic by ca. 0.25 eV). Thus, for both water and acetonitrile solvents, we investigated the acidity of two acid sites: the Al-O-Si bridge site, and the specific acid site. No such proton transfer was observed with dioxane solvent in the pore and thus only proton transfer from the bridge acid site to pyridine was investigated in this case (Supplementary Figure 35). All calculated energies are provided in Supplementary Table 3.

The first observation we make is that dioxane and water increase the acidity of the Al-OH-Si bridge, with calculated PTEs of -0.85 and -1.39 eV for dioxane and water, respectively, compared with -0.67 eV for the dry zeolite. In the case of acetonitrile, however, the acidity of the bridge depends on the solvent loading. At low acetonitrile loadings (6 molecules per unit cell), we see an increase in the acidity of the bridge (PTE = -0.93 eV), but at higher loadings, we see a drop in acidity, as the calculated PTE increases to ca. -0.5 eV for 9 and 10 molecules per unit cell. Electrostatics does not appear to be the factor that determines the acidity of the bridge site for these three solvents, as the dipole moments of acetonitrile, water and 1,4-dioxane are 3.92, 1.86 and 0 Debye, respectively. Competitive coordination around the adsorbate and the bridge site as well as van der Waals interactions seem to be playing an important role, too.

However, as we noted earlier, in water and acetonitrile, the thermodynamically favored acid site is no longer the bridge site, but rather the specific acids that form when the bridge proton facilely transfers to a solvent molecule (Supplementary Figures 36 and 37). This has a profound

effect on the effective acidity of the zeolite. Based on the computed PTEs, the acidity increases in the order water < dry zeolite < acetonitrile < dioxane (respective pyridine PTEs, -0.43, -0.67, -0.70 and -0.85 eV). The water clusters stabilize the proton so strongly that transfer to pyridine is more difficult than from the bridge site of the dry zeolite to the tune of 0.24 eV, which at 298 K amounts to about 4 orders of magnitude decrease in acidity. On the other hand, 1,4-dioxane increases the acidity of the zeolite by about 3 orders of magnitude, at 298 K. For a loading of 10 molecules per unit cell, acetonitrile lies in-between, increasing the acidity by about a factor of 3. We have investigated the effect of loading by varying the number of acetonitrile molecules from 6 to 10 per unit cell. Interestingly, the PTE to pyridine increases (acidity decreases) almost linearly until we reach a plateau at a loading of 9 molecules/unit cell. At lower acetonitrile loadings (6-7 molecules/unit cell), we have significantly stronger acidity (PTE of -1.29 and -1.08 eV, respectively) than in dioxane. At such loadings, after formation of the protonated acetonitrile dimer, the solvent molecules are distributed rather uniformly in the channels of the zeolite. As we push more solvent into the zeolite, we reach what seems to be the maximum stabilization of the proton by having 3 molecules coordinated around it. This seems to be attained at the loading of 9 molecules/unit cell. Further increase in the loading does not lead to further stabilization of the proton of the specific acid site.

Supplemental References

1. Sandler, S. I. *Chemical, Biochemical, and Engineering Thermodynamics*. (John Wiley & Sons, Inc., 2006).
2. Madon, R. J. & Iglesia, E. Catalytic reaction rates in thermodynamically non-ideal systems. *J. Mol. Catal. A Chem.* **163**, 189–204 (2000).
3. Eckstein, S., Hintermeier, P. H., Zhao, R., Baráth, E., Shi, H., Liu, Y. & Lercher, J. A. Influence of Hydronium Ions in Zeolites on Sorption. *Angew. Chemie - Int. Ed.* **58**, 3450–3455 (2019).
4. Latour, R. A. The Langmuir isotherm: A commonly applied but misleading approach for the analysis of protein adsorption behavior. *J. Biomed. Mater. Res. - Part A* **103**, 949–958 (2015).
5. Gould, N. S. & Xu, B. Temperature-Programmed Desorption of Pyridine on Zeolites in the Presence of Liquid Solvents. ACS Catal. 8, 8699–8708 (2018).
6. Liu, Y., Vjunov, A., Shi, H., Eckstein, S., Camaioni, D. M., Mei, D., Baráth, E. & Lercher, J. A. Enhancing the catalytic activity of hydronium ions through constrained environments. *Nat. Commun.* **8**, 14113 (2017).
7. Krossner, M. & Sauer, J. Interaction of Water with Bronsted Acidic Sites of Zeolite Catalysts. Ab Initio Study of 1:1 and 2:1 Surface Complexes. *Phys. Inorg. Chem.* **100**, 6199–6211 (1996).
8. Trombetta, M., Armaroli, T., Alejandre, A. G., Solis, J. R. & Busca, G. An FT-IR Study of the Internal and External Surfaces of HZSM5 Zeolite. *Appl. Catal. A Gen.* **192**, 125–136 (2000).
9. Trombetta, M. *et al.* FT-IR Studies on Light Olefin Skeletal Isomerization Catalysis. *J. Catal.* **179**, 581–596 (1998).
10. Vener, M. V., Rozanska, X. & Sauer, J. Protonation of water clusters in the cavities of acidic zeolites: (H2O) n ·H-chabazite, n = 1-4. *Phys. Chem. Chem. Phys.* **11**, 1702–1712 (2009).
